# The New Era of Pulmonary Hypertension: The Dawn of Disease Modification & Therapeutic Modalities

**DOI:** 10.3390/jcdd13050174

**Published:** 2026-04-22

**Authors:** Noyan Ramazani, Lacey Barnes, Alex Wong, Divyansh Sharma, Aditi Singh, KaChon Lei

**Affiliations:** 1 Department of Internal Medicine, Kirk Kerkorian School of Medicine, University of Nevada, Las Vegas, NV 89106, USA; lacey.barnes@unlv.edu (L.B.); alex.wong@unlv.edu (A.W.); aditi.singh@unlv.edu (A.S.); 2 Department of Cardiovascular Medicine, Kirk Kerkorian School of Medicine, University of Nevada, Las Vegas, NV 89106, USA; divyansh.sharma@unlv.edu

**Keywords:** pulmonary hypertension, pulmonary arterial hypertension, interstitial lung disease, chronic obstructive pulmonary disease, right ventricular systolic pressure, heart failure, sudden cardiac arrest, arrhythmias, chronic thromboembolism, phosphodiesterase inhibitors, sotatercept

## Abstract

Pulmonary hypertension (PH) can be defined as a mean pulmonary artery pressure (mPAP) greater than 20 mm Hg at rest during right heart catheterization (RHC). The reported prevalence of PH throughout the globe has been estimated to impact approximately 1% of the total population, with a majority of those afflicted being women more than men. Numerous etiologies give rise to the pathophysiology of PH, including heart disease (i.e., left-sided heart failure), lung diseases, and other unclear causes related to chronic stages and complications surrounding long-standing pulmonary thromboembolisms, side effects of certain medications, and genetic and environmental factors. Untreated PH can lead to severe morbidities such as cardio-renal syndrome and congestive hepatopathy (cardiac cirrhosis). Management of PH focuses on decreasing pulmonary pressures by using vasodilators such as prostanoids, and phosphodiesterase type 5 (PDE-5) inhibitors, as well as newer treatments such as sotatercept, which inhibits activin signaling, thereby inhibiting excessive cell growth in the pulmonary artery vasculature and down-regulating the pro-proliferative pathways.

## 1. Introduction

**Figure 1:**
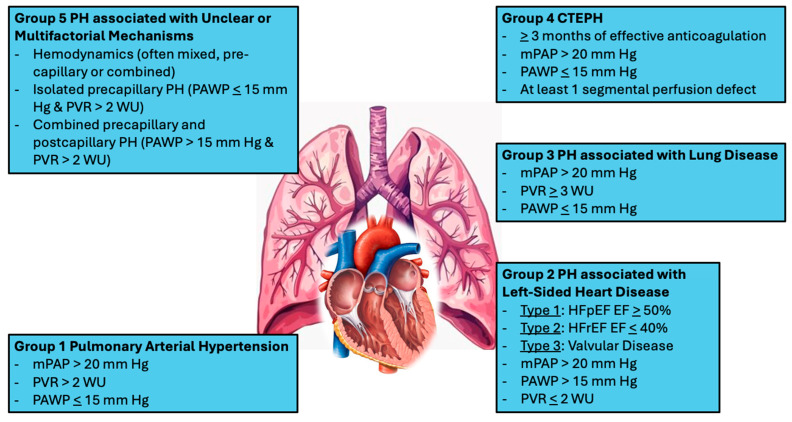
Diagnostic hemodynamic findings associated with the five groups of pulmonary hypertensions. **mPAP** = mean pulmonary arterial pressure; **PVR** = pulmonary vascular resistance; **WU** = Wood unit; **PAWP** = pulmonary artery wedge pressure; **PH** = pulmonary hypertension; **HFpEF** = heart failure with preserved ejection fraction; **HFrEF** = heart failure with reduced ejection fraction; **CTEPH** = chronic thromboembolic event pulmonary hypertension.

Pulmonary hypertension (PH) is a progressive and complex cardiopulmonary disorder that has historically been defined by elevated pulmonary arterial pressures (PAP) and associated with substantial morbidity and mortality. There are five main groups of pulmonary hypertension which are categorized as follows: pulmonary arterial hypertension (PAH) (rare), pulmonary hypertension (PH) associated with left-sided heart disease, PH associated with lung disease, PH related to thromboembolic disease or events often called chronic thromboembolic PH (CTEPH), and lastly PH with unclear and/or multifactorial mechanisms (Figure 1) [1]. Advances in invasive hemodynamic assessment have refined PH diagnostic criteria, with PH now defined by a mean pulmonary arterial pressure (mPAP) greater than 20 mm Hg at rest as measured by right heart catheterization (RHC), which remains the diagnostic gold standard [2,3]. While this updated definition has improved sensitivity for earlier disease detection, it has also highlighted an important reality: pulmonary hypertension is not a singular hemodynamic abnormality, but rather a heterogeneous disease process that evolves and manifests across a broad spectrum of clinical and biological phenotypes.

Over the past two decades, increasing awareness of PH among clinicians, combined with advances in non-invasive imaging, screening algorithms, and risk stratification tools, has led to earlier disease recognition across diverse populations. These efforts have revealed that PH is far more prevalent than previously appreciated, affecting approximately 1% of the global population, with prevalence rising sharply with advancing age [4,5]. Importantly, earlier detection has illuminated the often indolent and slowly progressive nature of PH. Many patients experience a prolonged subclinical phase characterized by subtle hemodynamic abnormalities and evolving pulmonary vascular remodeling long before overt right ventricular (RV) failure or advanced symptoms develop. PH causes pathologic remodeling with intimal hyperplasia, medial hypertrophy, adventitial proliferation, and the formation of plexiform lesions [6]. This extended disease trajectory presents a critical window for intervention, while simultaneously challenging clinicians to distinguish early pathologic disease from benign or transient elevations in pulmonary pressure.

Despite meaningful progress in diagnosis and disease awareness, late-stage pulmonary hypertension remains exceptionally difficult to treat. Advanced disease is marked by progressive pulmonary vascular remodeling, right ventricular maladaptation, and multisystem involvement, including hepatic, renal, and neurohormonal dysfunction [7]. Various organ systems can become adversely affected by persistent PH, including the eyes, thyroid, skin, brain, gastrointestinal tract, and skeletal muscles [7,8,9]. Once right ventricular failure and end-organ congestion are established, therapeutic responses are often attenuated, treatment options become limited, and mortality increases substantially [8,10]. These observations underscore the central importance of early identification and timely intervention, before irreversible vascular and myocardial changes dominate the clinical course.

Concurrently, expanding insights into the molecular, genetic, and immunologic underpinnings of pulmonary hypertension have fundamentally reshaped its conceptual framework. PH is now recognized as a biologically heterogeneous and multisystem disorder, encompassing distinct pathophysiologic mechanisms across its clinical subgroups [11,12]. Advances in genetics, epigenetics, and cellular signaling have revealed shared and divergent pathways driving vascular remodeling, inflammation, and maladaptive repair, providing a mechanistic foundation for targeted therapeutic development [13,14]. These insights have catalyzed the emergence of novel treatment strategies that extend beyond traditional vasodilator therapies, including antiproliferative and pathway-specific agents that aim to modify disease progression rather than solely alleviate symptoms.

As earlier diagnosis converges with biologically informed phenotyping and an expanding therapeutic armamentarium, a new horizon is emerging in the management of pulmonary hypertension. This evolving landscape offers the potential to shift PH from a disease predominantly managed in its advanced stages to one identified and treated earlier along its trajectory, to alter long-term outcomes. In this review, we examine pulmonary hypertension through the lens of growing disease awareness, early detection, and mechanistically targeted therapy. We synthesize contemporary classification, pathophysiology, diagnostic modalities, and emerging treatments to highlight how advances in understanding are reshaping the clinical approach to this complex and historically devastating disease.

## 2. Materials and Methods

We conducted a thorough search in medical databases such as PubMed, Embase, and Google Scholar for biomedical research articles, pertinent clinical trials, and analytical data from respective and reputable international governing bodies including but not limited to the American Heart Association (AHA), American College of Cardiology (ACC), the European Society of Cardiology (ESC), and the European Respiratory Society (ERS). Our goal was to investigate applicable clinical registries on pulmonary hypertension (PH) and related keyword variants. All compiled data was selected between the timeframe in the past 30 years (1996–2026) to highlight the evolution of this disease and the scientific breakthroughs in identification, diagnosis, gauging prognostic implications through imaging modalities, and lastly providing accessible treatment options and management strategies.

## 3. Epidemiology

Contemporary epidemiologic data increasingly reflect a broader disease burden and a growing population of patients living with PH across its various subtypes. Improved clinical awareness, advances in diagnostic strategies, and earlier detection have shifted PH toward recognition as a chronic, progressive disease in which disease severity, risk, and clinical trajectory evolve over time.

In Western countries, pulmonary arterial hypertension (PAH) affects approximately 25 individuals per 1 million population, with a higher prevalence among women [15]. Even with the development of specific medications for PAH, the mortality rate of the disease is still elevated. Between 1990 and 2021, the global prevalence of PAH remained higher in women than men, but men experienced greater reductions in mortality and disability-adjusted life years (DALYs), leading to a reversal whereby in 2021, men had lower DALYs than women [16]. Furthermore, in 2021, the age-standardized prevalence of PAH was highest in high-sociodemographic index (SDI) regions and lowest in low-middle SDI regions, whereas mortality rates peaked in middle-SDI regions and were lowest in high-SDI regions [16]. Chang et al. used data from the Pulmonary Hypertension Association (PHA) Registry (2015–2020) and found that while overall 3-year mortality in PAH patients was 21%, the outcomes varied by risk group, and high-risk patients faced up to 55% mortality [17]. This highlights the importance of early screening and diagnosis in this population. 

Idiopathic PAH is a rare form of pulmonary hypertension. Data from The REVEAL registry and the French registry showed that idiopathic PAH accounted for 46% of 2525 patients and 39% of 674 patients, respectively [3]. Connective tissue disease-associated pulmonary arterial hypertension (CTD-PAH) is the second most common form of PAH after idiopathic PAH. Systemic sclerosis accounts for 75% of cases of CTD-PAH, followed by systemic lupus erythematosus (SLE) and mixed connective tissue disease (MCTD) [18]. REVEAL registry data shows geographic variation in CTD-PAH. With systemic sclerosis pulmonary arterial hypertension (SSc-PAH) predominating in the UK, while SLE-PAH is more common in Chinese and Korean populations [18]. A Taiwanese study using the national database (2002–2013) found that while SLE accounted for the majority of CTD-PAH cases, SSc carried the highest risk and worst survival [19]. 

Heritable PAH affects around 6–10% of patients with PAH, and variants in the Bone Morphogenic Protein Receptor Type 2 (BMPR2) have been found to account for almost 75% of heritable PAH cases with an autosomal dominant pattern [3]. The incidence and prevalence of PAH associated congenital heart disease (CHD) is 2.2 and 15.6 per 1 million, respectively [20]. Among 192 patients with CHD-PAH, 20-year survival was highest in those with Eisenmenger syndrome (87%) or systemic-to-pulmonary shunts (86%), but markedly lower in patients with corrected defects such as atrial or ventricular septal defects (36%) [3]. When measured by echocardiography, the prevalence of pulmonary hypertension is greater in HIV patients (2.6–14%) compared to the general population (1%) [21]. A study by Salvador et al. used the Spanish PAH registry (REHAP) and compared the data to a matched cohort with idiopathic/familial PAH. The data showed that patients with HIV-PAH were younger and less likely to be female [21].

## 4. Risk Factors

Understanding the risk factors for pulmonary arterial hypertension associated with certain conditions facilitates earlier recognition of high-risk patients, more timely screening, prompt initiation of treatment, and improved survival. This section will focus on the risk factors for PAH associated with various underlying conditions. 

Jiang et al. conducted a systematic review to explore the association of systemic sclerosis clinical characteristics, antibodies, labs and biomarkers with PAH, and found that the most frequent associations were low diffusion capacity of the lung for carbon monoxide (DLCO), older age, positive anticentromere antibodies, presence of telangiectasias, and elevated brain natriuretic peptide (BNP) [22]. Notably, certain factors associated with SSc-PAH could also be manifestations of the pulmonary hypertension itself. For example, an elevated BNP could be due to increased right heart strain secondary to PAH. Another study via Didriksen et al. assessed serum levels of vascular endothelial growth factor C (VEGF-C) and angiopoietin 2 (Ang-2) in systemic sclerosis patients with and without PAH, and found that SSc-PAH patients had lower mean serum levels of VEGF-C and higher mean serum levels of Ang-2 [23]. 

This supports the hypotheses that lymphangiogenesis is deregulated during PAH development in SSc and indicates that VEGF-C could be a marker for early PAH in this patient population [23]. Another potential risk factor of SSc-PAH is autoimmunity against G Protein-Coupled Receptors (GPCRs). Gluschke et al. explored the Sphingosine-1-phosphate receptor (S1PR) as an antigen for PAH autoimmunity. Autoantibodies against S1PR2 and S1PR3 were more common in those with PAH (25.9% and 27.6%, respectively) than in healthy controls (<10%), which suggests that these receptors may act as autoantigens and warrants further investigation [24].

A meta-analysis via Lun et al. assessed the risk factors for SLE-PAH and found that patients with SLE have a higher risk of developing PAH when presenting with Raynaud’s phenomenon, anti-ribonucleoprotein (anti-RNP) antibody positivity, interstitial lung lesions, serositis, pericardial effusion, vasculitis, or rheumatoid factor positivity [25]. Notably, anti-double-stranded DNA antibodies (anti-ds-DNA Ab) and anti-smith antibodies (anti-sm Ab) were not found to be clinically significant risk factors associated with SLE-PAH [25]. Furthermore, another meta-analysis by Liu et al. determined that female gender, alopecia, systemic hypertension, anti-Sjögren’s syndrome type b autoantibodies (anti-ssb auto-Ab), anti-ssb antibody positivity, anti-U1 small nuclear ribonucleoprotein particle (anti-U1RNP) antibody positivity, thrombocytopenia, and current smoking status were all risk factors associated with SLE-PAH [26]. An evolving area of research in this patient population is the role that the atypical activation of the immune system plays in the development of SLE-PAH. A recent study via Li et al. identified inflammation, especially interferon and interleukin-6 (IL-6) signaling, as a central driver of SLE-PAH, with distinct molecular subgroups and potential therapeutic targets such as Kruppel-like factor 1 (KLF1) and GATA binding protein 1 (GATA1) [27]. 

In terms of the characterization of patients with MCTD-PAH, Chaigne et al. conducted a study comparing patients with MCTD-PAH and MCTD patients without PAH and found that pericarditis, polyarthritis, thrombocytopenia, interstitial lung disease (ILD), and anti-Sm antibody positivity were risk factors for MCTD-PAH [28]. Recent studies have identified autoantibodies associated with MCTD-PAH. Thoreau et al. performed a multi-center study including CTD patients with and without PAH and found that the anti-Annexin A5 IgG was significantly more frequent in MCTD patients with PAH compared to those without PAH [29]. In a study of the association of severe PAH and systemic autoimmune rheumatic diseases, specifically SLE, rheumatoid arthritis (RA), SSc, inflammatory myopathies, and primary Sjögren’s syndrome, data suggested that comorbid ILD, heart failure, valvular disease, and use of steroids or immunosuppressants may increase the risk of severe PAH [30]. 

Studies have shown that there is a potential for elevated risk of Sjögren’s syndrome-PAH (SS-PAH) in male patients. Coppi et al. conducted a study comparing male and female patients with SS, and found that male patients had lower respiratory function, greater dilatation and signs of diastolic dysfunction, increased pulmonary artery diameter, and higher right ventricular outflow tract time-velocity integral [31]. There have been a limited amount of studies assessing the genetic susceptibility in SS-PAH. A study of 34 patients with primary SS-PAH identified 141 pathogenic variants across 129 genes, with five key candidate mutations, including in genes *FLG*, *BCR*, *GIGYF2*, *ITK*, and *SLC26A4* [32]. This could suggest the potential for genetic markers for early detection. 

Among patients that are eligible for liver transplantation, the prevalence of portal hypertension-associated pulmonary hypertension (PoPH) ranges from 4 to 5%, with autoimmune hepatitis and female sex being identified as risk factors [33]. Furthermore, there is no known association between the severity of liver disease or degree of portal hypertension and the development of PoPH [34]. In terms of potential risk factors for HIV-associated PAH, Duncan et al. conducted a study to determine risk factors of PAH in veterans with HIV, and found that a low CD4 count and a high HIV viral load contributed to an increased risk of PAH in the patient population [35]. This supports the notion that effective control of HIV should be emphasized to reduce the risk of pulmonary hypertension in this population. 

## 5. Genetics

PAH is manifested by both a genetic and environmental component, and despite recent therapeutic advances, it is still considered a rare disease with high mortality amongst the populations and demographics of people that it inflicts. PAH is known to have a gene-environment interaction in which the genetic framework and blueprint of the disease are modified by environmental exposures and triggers, thereby worsening or exacerbating the disease process [14]. The rudimentary cellular and molecular etiology of PAH is caused by the occlusion of the pulmonary arterioles rooted in the dysfunction of endothelial cells and uninhibited regulation of both fibroblasts and pulmonary smooth muscle cell proliferation [13]. 

Genetically, PAH is typically caused by autosomal dominantly inherited genes such as *BMPR2*, which is the major gene causing familial forms of PAH (FPAH) while the environmental risk factors such as hypoxia, and exposure to drugs and toxins and epigenetic factors like active histone marker H3K27ac are contributory [14]. While genetics plays a huge role in PAH in adults, it heavily impacts the pediatric population and causes severe clinical comorbidities affecting both lung and heart development pathologies. The two most notable causal genes with the highest frequencies of deleterious effects among the pediatric population include the *TBX4* and *SOX17* genes which are expressed in embryonic tissues and possess vital roles in both lung and vasculature development [14].

The discovery of gene transcription and translation regulatory factors such as T-box TF 4 and SRY-box TF 9 had previously been discovered in only the prenatal pediatric population, but recent research has described these epigenetic regulators to become reinduced or repression deactivated and have been described to cause persistent pulmonary hypertension (PH) in newborns and PAH in adults [12,36]. Chelladurai et al. and colleagues worked on silencing developmental transcription factor expression in TBX4, TBX5, and SOX9, as well as EP300, and discovered that this act led to the regression of mesenchymal signatures, and reduction in vascular remodeling causing improved hemodynamics and reduced muscularization of the distal pulmonary arteries [36].

Alterations in genes regulating DNA methylation have also been linked to the development of PAH with somatic mutations of the Tet Methylcytosine-Dioxygenase-2 (*TET2*) gene, which is vital for removing methyl groups, from cytosine nucleotides in DNA [12,37]. Recently, *TET2* gene mutations have been identified to be associated with inflammation, atherosclerosis, pulmonary vascular obliteration, and pulmonary hypertension (PH) [37]. Acquired mutations in the *TET2* gene have also been implicated in the discovery of clonal hematopoiesis of indeterminate potential (CHIP), which is a precursor for myelodysplastic syndrome (MDS), myeloproliferative neoplasms, and even acute myeloid leukemia (AML) [37].

Yan et al. showed that DNA methyltransferase 3B (DNMT3B), which is crucial for DNA methylation, was upregulated in both PH patients and rodent models and inhibition of DNMT3B promoted proliferation of pulmonary artery smooth muscle cells (PASMCs) in response to platelet-derived growth factor-BB (PDGF-BB), while overexpression improved hypoxia-mediated PH and right ventricular hypertrophy (RVH) in mice models [38]. Extensive studies on PASMCs have shown that excessive proliferation and impaired apoptosis pathways of PASMCs are contributory to vascular obstruction in both patients and rodents with PAH [39]. In addition, the molecular activity and genetic expression of the mitochondrial superoxide dismutase-2 (SOD2) enzyme, which is the major generator of hydrogen peroxide (H_2_O_2_), is reduced in PAH [39].

The 16 common genes (Table 1) that have been implicated in the diagnosis and etiology of PAH [13]. This is by far not an all-inclusive list as new research studies, clinical developments, and the invention of advanced therapeutic modalities have caused the discovery of new genetic markers to be detected in the disease process of PAH. In addition, it is notable to know that the genes mentioned below offer variable penetrance patterns that will underscore the critical expression of specific genes leading to the known consequential functional roles when mutations have occurred.

## 6. Classifying the Five Groups of Pulmonary Hypertension

### 6.1. Group I Pulmonary Arterial Hypertension

Pulmonary arterial hypertension (PAH) pertains to group 1 of the pulmonary hypertension classification (Table 2). This is diagnosed through a right heart catheterization (RHC). The definition of pulmonary hypertension through RHC was changed in 2022 to focus on early detection of the disease and to prevent delayed diagnosis, which is linked to elevated morbidity and shortened lifespan [40]. The original definition of pulmonary arterial hypertension utilized a mean pulmonary artery pressure (mPAP) ≥ 25 mm Hg, pulmonary vascular resistance (PVR) > 3 Wood units (WU), and pulmonary arterial wedge pressure (PAWP) ≤ 15 mm Hg, but studies have shown that there is a significant increase in mortality and hospitalization with mPAP > 20 mm Hg [40]. Deviations from these numbers arising from lack of medical therapy or intervention can cause deleterious health effects. Thus, newer diagnosis cut-off suggests a mPAP > 20 mm Hg.

Research has also shown that a mildly elevated PVR leads to elevated mortality and worse prognosis; therefore, the updated definition of pulmonary arterial hypertension uses an mPAP > 20 mm Hg, PVR > 2 WU, and PAWP ≤ 15 mm Hg [42]. PH can be subclassified into 3 groups: isolated precapillary PH (PAWP ≤ 15 mm Hg and PVR > 2 WU), isolated postcapillary PH (PAWP > 15 mm Hg and PVR ≤ 2 WU) and combined precapillary and postcapillary PH (PAWP > 15 mm Hg and PVR > 2 WU). Hemodynamically, pulmonary arterial hypertension (Group 1 PH) is classified as precapillary PH [41]. 

Group 1 pulmonary hypertension can also be subcategorized into different etiologies: idiopathic PAH (IPAH), heritable PAH (HPAH), connective tissue disease (CTD)-associated PAH, congenital heart disease-associated PAH, drug-and-toxin-induced PAH, HIV-associated PAH, PAH associated with portal hypertension, persistent pulmonary hypertension of the newborn, and infection-induced PAH. 

#### 6.1.1. Idiopathic Pulmonary Arterial Hypertension

Idiopathic PAH (IPAH) is a rare and progressive lung disease that is believed to have a combination of both genetic and environmental factors leading to the conclusion that epigenetics has a large influence in IPAH. IPAH refers to an elevated mPAP and PVR in the absence of other etiologies such as connective tissue disease, chronic hypoxia, and heart disease. It is considered a diagnosis of exclusion, and is the most common form of PAH [43]. One distinct phenotype of IPAH is the lung phenotype, characterized by a smoking history and a low lung diffusion capacity for carbon monoxide (DLCO) with no other signs of lung disease; these patients are considered separate from patients with group 3 PH [43]. 

#### 6.1.2. Heritable Pulmonary Arterial Hypertension

Several studies have demonstrated a genetic component in the development of pulmonary arterial hypertension, and approximately 3% of all PAH patients are classified as heritable PAH (HPAH) [44]. HPAH is most commonly caused by heterozygous variants in the *BMPR2* gene, which predisposes to narrowing of the small pulmonary arteries secondary to cell proliferation and prevention of apoptosis, leading to vascular remodeling [44]. 

#### 6.1.3. Pulmonary Arterial Hypertension Associated with Connective Tissue Disease

Another association of PAH is with connective tissue disease (CTD). Pathologies included in the category of PAH-CTD includes systemic sclerosis (SSc), systemic lupus erythematosus (SLE), mixed connective tissue disease (MCTD), Sjögren’s syndrome (SS), idiopathic inflammatory myopathies (IIM), and rheumatoid arthritis (RA). Within CTD-associated PAH, systemic sclerosis is the leading cause of PAH in Western countries and SLE and mixed connective tissue disease are associated with PAH in Asia [41]. 

##### Systemic Sclerosis

PAH in systemic sclerosis (SSc) is known as a severe complication of the disease, and it occurs in 8–12% of SSc cases, seen through a RHC procedure [41]. The complexity of SSc-PAH lies in the prevalence of restrictive interstitial lung disease (ILD), pulmonary veno-occlusive disease (PVOD), and/or left ventricular (LV) diastolic dysfunction (LVDD) in the setting of systemic sclerosis, making SSc a contributor to multiple groups of PH. In SSc-ILD, the progressive fibrosis, inflammation, and vascular injury results in replacement of the normal pulmonary architecture with scarring, leading to subsequent dyspnea, non-productive cough, and fatigue as the most common symptoms [45]. Systemic sclerosis is associated with an increased risk of developing a phenomenon called pulmonary veno-occlusive disease (PVOD), in which vascular remodeling of pulmonary veins and venules leads to fibrotic occlusion. This is represented by congestion of capillaries and alveolar hemorrhage and is illustrated by lymph node enlargement, centrilobular ground-glass opacities, and septal lines on a high-resolution chest computed tomography (HRCT) imaging study [41]. 

SSc has an increased prevalence of left ventricular diastolic dysfunction (LVDD), through a mechanism of myocardial fibrosis, microvascular disease, and chronic inflammation, resulting in decreased ventricular relaxation and increased filling pressures [46]. Oftentimes, in patients that have been diagnosed with SSc, PAH is diagnosed at the later, more severe stages of the disease, complicated by severe hemodynamic impairment [41]. Furthermore, PAH accounts for approximately 30% of deaths related to SSc, so early screening for PAH in these patients is very important and can have a positive impact on prognosis [41]. 

There is a recommended annual screening strategy for PAH in SSc, which has been composed of a combination of clinical presentation, biomarkers in the blood, pulmonary function tests (PFTs), and echocardiographic features. The trajectory of PFTs over time has been shown to be a tool for not only diagnosis, but assessment of severity of pulmonary dysfunction in a patient with SSc. An analysis by Nihtyanova et al. showed patients with diffuse cutaneous SSc showed a decline in the diffusing capacity of carbon dioxide (DLCO) and the carbon monoxide transfer coefficient (KCO) of 1.5% per year in all patients but demonstrated a greater decline annually (4.5% and 4.8%) in the 5–7 years before diagnosis of PH [47].

The DETECT algorithm is used to screen for PAH in patients with SSc lasting more than 3 years, effectively excluding early cases. These patients must have a forced vital capacity (FVC) of less than 40% and a DLCO below 60% [48]. The first step of the algorithm calculates a risk score using factors such as telangiectasias, anti-centromere antibodies, NT-proBNP levels, uric acid levels, electrocardiogram (EKG) findings, and FVC/DLCO ratio. If this score is above 300, the next step assesses cardiac measurements with echocardiography, specifically the right atrial area and tricuspid regurgitation velocity. The final score from both steps helps to determine whether a patient should undergo a RHC procedure [48].

##### Systemic Lupus Erythematosus

Systemic lupus erythematosus (SLE) can lead to multiple groups of pulmonary hypertensions, including PH associated with interstitial lung disease, PAH, and chronic thromboembolic pulmonary hypertension (CTEPH) [18]. Although SLE is more prevalent than SSc overall, SLE-associated pulmonary hypertension is less common than systemic sclerosis-associated pulmonary hypertension. The majority of CTD-PAH patients of Asian descent have SLE-PAH, and the mortality rate exceeds 14% [42]. Predictive factors of PH in SLE have been shown to be younger patient age, presence of anti-smooth muscle antibodies (ASMAs) or anticardiolipin antibodies (ACLAs), and positive history of pericarditis [18].

##### Mixed Connective Tissue Disease

Mixed connective tissue disease (MCTD) can be associated with features of systemic sclerosis and systemic lupus erythematosus and can have lung and pulmonary vasculature involvement [18]. MCTD accounts for around 9% of CTD-PAH [49]. According to the current guidelines, PAH screening is recommended in asymptomatic patients with features of systemic sclerosis, which includes patients with MCTD. 

##### Rheumatoid Arthritis

Rheumatoid arthritis (RA) is a chronic systemic inflammatory disease that primarily affects the synovial joints and can also involve the extra-articular organs, specifically the lungs. In the older REVEAL study completed in the US in 2012, RA-associated PAH was reported in 8–9% of patients with CTD-PAH, and showed a better survival rate compared to patients with SSc-PAH [49]. Current guidelines do not suggest PAH screening of asymptomatic patients with RA.

##### Sjögren’s Syndrome

Primary Sjögren syndrome (pSS) is a chronic systemic autoimmune disease that has a rare association with pulmonary arterial hypertension. The largest case report series reported pSS in only 9 patients with PAH; however, it accounts for 15–16% of CTD-PAH cases in Chinese registries [49]. 

##### Idiopathic Inflammatory Myopathies

Idiopathic inflammatory myopathies (IIM) such as dermatomyositis (DM) and polymyositis (PM) have been shown to have a rare association with pulmonary arterial hypertension; however, in these studies, causes of PH such as group 3 PH due to chronic hypoxia could not be excluded [49]. Dermatomyositis is an idiopathic inflammatory myopathic disease that can have pulmonary involvement, specifically with ILD or PH. Wang et al. analyzed clinical characteristics of patients with DM complicated by pulmonary hypertension and found that DM-PH had a higher IL-6, IL-10, and lower IL-17, double-positive (DP) cell ratio, and B-lymphocyte ratio than that of non-DM-PH making pro-inflammatory chemical markers present in higher concentrations in IIM compared to non-IIM associated with PH [50]. 

#### 6.1.4. Pulmonary Arterial Hypertension Associated with Congenital Heart Disease

Patients with congenital heart disease (CHD)-associated pulmonary hypertension can be subcategorized into 4 different categories: group 1 PAH, group 2 (Left Heart Disease), group 4 (Chronic Thromboembolic Pulmonary Hypertension), group 5 (Unclear/Multifactorial mechanism) [20]. Etiologies of group 1 (PAH) include Eisenmenger syndrome, left-to-right shunt, PAH associated with a small defect, and PAH after a CHD correction. Etiologies of group 2 PH include pulmonary vein stenosis, ventricular dysfunction, valvular disease, and left heart obstructive disease. Group 4’s etiology includes pulmonary artery obstruction due to congenital pulmonary artery stenosis. Etiologies of group 5 include a single ventricle or segmental pulmonary hypertension [20]. Classification of a patient into one specific group based on clinical presentation to guide individualized management is complex; patients could develop characteristics of multiple different groups over time.

Eisenmenger syndrome (ES) is a type of congenital heart disease defined by a severe elevation of PVR, resulting in the reversal of the direction of a shunt from a systemic-to-pulmonary pathway to a pulmonary-to-systemic pathway, leading to chronic hypoxemia [20]. ES comprises 58% of adult congenital heart disease (ACHD)–PAH patients, and ventricular septal defect (VSD) is the most common underlying abnormality and is seen in 42% of patients [51]. The risk of mortality in this population is elevated, even in patients treated with pulmonary vasodilators. 

In a study of patients with ES treated with pulmonary vasodilator therapies, mortality was 8.4% per year in untreated patients and 4.8% per year in treated patients [51]. Moderate-to-large defects associated with a left-to-right shunt typically lead to a mild-to-moderate elevation in PVR and are grouped by repairability based on the severity of PVR elevation. Patients with a mild PVR of < 4 WU are considered repairable, patients with a PVR > 8 WU are considered unrepairable, and patients with an intermediate PVR (4–8 WU) are typically considered for targeted PH therapy [20]. Development of PAH after surgical repair of CHD has been associated with poor prognosis, and there are currently no reliable predictors of which patients are at risk for developing this condition [20]. These patients typically do not have cyanosis or a blood shunt, and clinical presentation is similar to idiopathic pulmonary hypertension [51]. 

#### 6.1.5. Drug-and-Toxin-Induced Pulmonary Arterial Hypertension

A clinically significant subgroup of group 1 PH is drug-and-toxin-induced pulmonary arterial hypertension. Certain chemical agents have been deemed to have a “definite” association with pulmonary hypertension through outbreaks, case–control studies, and large multicenter series, and these include Aminorex, Fenfluramine, Dexfenfluramine, Benfluorex, methamphetamine, Dasatinib, and toxic rapeseed oil [52]. Methamphetamines and amphetamine-like anorexigens such as Aminorex, Fenfluramine, Dexfenfluramine, and Benfluorex have been shown to cause PH through an increase in serotonin levels through an interaction with serotonin transporter (SERT) and increased pulmonary artery smooth muscle cell proliferation. Methamphetamine induced PAH (MA-PAH) has been studied extensively, and has been found to be readily distributed to the lungs, with positron emission tomography (PET) scans after injection of methamphetamine showing the highest uptake in the lungs and liver [53]. 

MA-PAH and IPAH have been compared in past studies, and although both are defined by a precapillary pulmonary hypertension, MA-PAH has shown to have distinct features. A study by Kolaitis et al. compared 118 patients with MA-PAH and 423 patients with IPAH and found that MA-PAH showed less favorable hemodynamics compared to IPAH, with higher PVR, lower cardiac output (CO), and lower stroke volume index (SVI) [53]. Dasatinib, a tyrosine kinase inhibitor, evokes pulmonary hypertension through generation of reactive oxygen species (ROS), endothelial dysfunction, and increased endothelial permeability [52]. Toxic rapeseed oil is derived from a plant possibly contaminated with anilides, and causes endothelial dysfunction and narrowing of pulmonary arteries, along with intimal proliferation, fibrosis, and thrombosis, causing pulmonary hypertension [52].

#### 6.1.6. Pulmonary Arterial Hypertension Associated with HIV

Pulmonary hypertension is a well-known complication of HIV with studies using echocardiography showing that the prevalence of HIV related PAH is anywhere from 2.6 to 27.8% [54]. The pathophysiology of HIV-PAH is multifactorial, and hypothesized to be caused by chronic inflammation, protein Gp120, transactivator of transcription (Tat) protein negative factor (nef), and genetic predisposition determined by HLA-DR6 and HLA-DR52 frequency [55]. Previous studies have shown that the development of PAH in patients with HIV is related to the length of HIV infection, but is independent of CD4 count, viral load, or history of opportunistic infections [55]. However, more recent studies are challenging prior hypotheses. 

A meta-analysis and systematic review by Liu et al. evaluated the risk factors for PAH in patients living with HIV and found that HIV patients with PAH had a higher mean age, lower mean CD4 count, detectable viral load, were more likely to have a history of intravenous (IV) drug use and were less likely to have received antiretroviral therapy [54]. Since the recommendations were updated in 2018, screening for PAH in HIV patients is considered the standard of care, specifically for patients with female sex, IV drug use or cocaine/methamphetamine use, hepatitis C virus infection, origin from a high-prevalence country, known Nef or Tat HIV proteins, or US African American patients independent of symptoms [55]. 

Studies have also shown that patients with HIV-PAH are at higher risk for poorer prognosis. Youssef et al. performed a meta-analysis and systematic review which determined that the mean survival of patients with HIV-PAH was 37.4 months and emergency department visits and hospitalization rates were 73% and 71% respectively, which emphasizes the importance of screening in this population [56]. 

#### 6.1.7. Pulmonary Arterial Hypertension Associated with Portal Hypertension

A significant complication of portal hypertension is pulmonary arterial hypertension, also referred to as Portopulmonary Hypertension (PoPH). This condition is most commonly seen in cirrhotic patients, but patients with non-cirrhotic portal hypertension can also develop it [57]. In terms of the pathophysiology of PoPH, this topic is currently undergoing active research, and there are multiple hypotheses that explain the etiology of this disease. Proposed mechanisms of blood flow obstruction in the pulmonary arteries include circulating estradiol and prostacyclin synthase deficiency, dysfunctional liver and portosystemic shunts exposing the pulmonary vasculature to harmful factors, especially in genetically susceptible individuals [57]. Studies have shown that PoPH has a poor prognosis. Mean survival with no intervention is approximately 15 months, with a 6-month survival rate of 50% and a 5-year survival rate of 4–14% [34]. 

#### 6.1.8. Persistent Pulmonary Hypertension of the Newborn

Persistent pulmonary hypertension of the newborn (PPHN) is another subset of group 1 pulmonary hypertension. This condition is reported in 0.2% of live births among term newborns and 2% of live births in preterm infants [58]. This disorder is due to extrapulmonary shunting of deoxygenated blood from right-to-left across the patent ductus arteriosus (PDA) and patent foramen ovale (PFO), leading to a sustained elevation of PVR and subsequent severe hypoxemia [59]. There are 3 types of PPHN, categorized by etiology. The three etiologies are maladaptation (pulmonary vessels have a normal structure and number but abnormal vasoreactivity), excessive muscularization (increased thickness of smooth muscle and increased extension of muscle to vasculature that is usually not muscularized), and underdevelopment (lung hypoplasia with decreased number of pulmonary arteries) [59]. 

#### 6.1.9. Infection-Induced Pulmonary Arterial Hypertension

Pulmonary arterial hypertension can occur secondary to other infectious etiologies other than HIV which was previously mentioned. Schistosomiasis, and the hepatitis C virus are two other infectious etiologies inducing pulmonary arterial hypertension should they persist without treatment intervention. 

Chronic schistosomiasis is a parasitic disease that, in its hepatosplenic form, can lead to PAH, referred to as schistosomiasis-associated PAH (Sch-PAH) [60]. Schistosomiasis is considered one of the leading causes of PAH worldwide due to its high prevalence, affecting around 200 million people globally [60]. One mechanism of the pathogenesis of Sch-PAH is an immune-mediated inflammatory cascade, resulting in vascular remodeling at the intimal level and medial thickening [61]. Another mechanism involves the schistosomiasis eggs that are produced by adult schistosoma worms. The egg’s presence in the portal venous system can evoke an inflammatory response, which results in granuloma formation and subsequent fibrotic destruction and formation of a portosystemic shunt, causing the eggs to embolize into the pulmonary circulation [60].

Hepatitis C virus-associated PAH is rare but has been documented in case reports. Zhao et al. presented a case of a patient with chronic hepatitis C who developed pulmonary arterial hypertension, along with nephrotic syndrome and polymyositis. The patient showed significant improvement in 6 min walking distance at 3-month follow up and decreased pulmonary artery pressure with Sildenafil and Macitentan treatment [62].

### 6.2. Group II Pulmonary Hypertension Associated with Left-Sided Heart Disease

Group 2 PH associated with left-sided heart failure and group 3 PH are both considered the most prevalent groups of PH, accounting for approximately 90–95% of all PH cases recorded globally [5]. Group 2 PH can be further classified into three distinct types: Type 1—PH due to heart failure with preserved ejection fraction (HFpEF) secondary to an ejection fraction (EF) of equal to or greater than 50% (EF ≥ 50%); Type 2—PH due to heart failure with reduced ejection fraction (HFrEF) secondary to an EF less than or equal to 40% (EF ≤ 40%); Type 3—PH due to a pure valvular heart disease (VHD) as being the main cause of dysfunction [11,63].

The diagnosis of group 2 PH has several key hemodynamic characteristic features that distinguish it from other groups. The general definition for group 2 PH related to left-sided heart disease (group 2 PH-LHD) is having an mPAP > 20 mm Hg and a PAWP > 15 mm Hg [2]. The post-capillary PH, often termed isolated post-capillary pulmonary hypertension (IpcPH), is defined by a PVR ≤ 2 WU and a combined pre- and post-capillary pulmonary hypertension (CpcPH) by PVR > 2 WU [2]. The previous hemodynamic metric variable, diastolic pressure gradient (DPG), calculated as the difference between diastolic pulmonary arterial pressure (dPAP) and pulmonary artery wedge pressure (PAWP), is no longer used to distinguish between IpcPH and CpcPH because newer conflicting data on its prognostication has shown weak values amongst LHD [2].

Group 2 PH causes the pulmonary artery to develop higher levels of resistance and back pressure due to the collapse of the normal hemodynamic mechanical components of the left-side of the heart [11]. Some pathological etiologies impacting left-sided heart disease which gives rise to group 2 PH include left ventricular (LV) systolic dysfunction, LV diastolic dysfunction, valvular heart disease caused by either stenosis or regurgitation, and lastly any left heart inflow and outflow obstructions which are not related to valvular disease or congenital cardiomyopathies. It is important to mention that all of these complications cause increase pressures on the left side of the heart which negatively affects the pulmonary artery due to elevations in vascular resistance which leads to group 2 PH associated with left-sided heart disease.

Group 2 PH is commonly studied and researched to arise as a consequence and complication of chronic left atrial hypertension in heart failure (HF) caused by myocardial and valvular disease [64]. The cardiac remodeling that occurs as a consequence of this process induces alterations in the pulmonary artery capillaries, venous circulation, and leads to the promotion of right ventricle (RV) dysfunction and right-sided heart failure thereby increasing morbidity and mortality in both patients with heart failure with preserved ejection fraction (HFpEF) and in those with heart failure with reduced ejection fraction (HFrEF) [64]. Further studies have investigated the structure and function of the pulmonic vasculature as it molds in order to compensate for the failing left-sided heart failure and reflexively initiates pulmonary arterial and venous remodeling with the severity of PH being associated with the development of venous and small arteriolar intimal thickening [63,65,66].

Among the group of patients with group 2 PH with a concomitant HF diagnosis, those individuals with HFpEF were older, less likely male, were more obese, and had higher prevalence of hypertension and atrial fibrillation than the patients in the HFrEF category that had group 2 PH [66]. The patients with PH due to HFpEF were also less likely to have smoked cigarettes but still had the similar prevalence patterns and association with lung disease, obstructive sleep apnea (OSA), and renal dysfunction when compared to patients with HFrEF [66]. Other research findings amongst HFpEF patients that had developed group 2 PH found that LV size was smaller, and while LV wall thickness and mass was greater, E/e’ ratio and the prevalence of left atrium (LA) and right ventricle (RV) enlargement and dysfunction were not significantly different between HFpEF and HFrEF [66].

In order to distinguish between group 1 PAH and group 2 PH-LHD, a fluid challenge can be used as a diagnostic tool to possibly help unmask occult group 2 PH-LHD [67]. This can be done by increasing the left-sided filling pressures via the process of infusing 500 cc of saline over the span of 5–10 min which will cause a rise in pulmonary artery wedge pressure (PAWP) to >18 mm Hg indicating a latent postcapillary PH [67]. This diagnostic modality is invaluable, especially if patients have a PAWP between 13 and 15 mm Hg. However, some research highlights the poor pretest probability of PH-LHD during a fluid challenge as it can cause a false-positive increase in pressure changes making group 2 PH-LHD more likely [67]. Thus, other experiments have utilized a lower cut-off of PAWP of >12 mm Hg during baseline hemodynamic determinations rather than a cut-off of >15 mm Hg to make the increase in left-sided filling pressures of >18 mm Hg more substantial and clinically significant [67].

### 6.3. Group III Pulmonary Hypertension Associated with Lung Disease

Group 3 PH associated with lung disease (LD) accounts for approximately 10% of all cases of PH and is the second most prevalent type of PH after group 1 PAH [68]. Group 3 PH LD is classically associated with chronic lung disease and hypoxia and is characterized by an elevation of pulmonary vascular pressure secondary to chronic obstructive pulmonary disease (COPD), interstitial lung disease (ILD), obstructive sleep apnea (OSA), other lung conditions such as cystic fibrosis (CF), and developmental lung diseases such as bronchopulmonary dysplasia (BPD), and lastly high-altitude exposure [68,69]. 

The association between disease severity amongst chronic lung disease and morbidity and mortality from PH exists. Patients that have uncontrolled chronic lung diseases and abstain from any medical therapy or intervention and possess frequent exacerbations and re-hospitalizations are at higher risk of developing group 3 PH and will develop poor health outcomes translating into having shorter lifespans from the time of chronic lung disease diagnosis to development of group 3 PH and the complications that stem from it that eventually leads to death [69]. 

Group 3 PH LD is hemodynamically defined as a precapillary PH with the following pressure gradient metrics: mean pulmonary arterial pressure (mPAP) > 20 mm Hg, pulmonary arterial wedge pressure (PAWP) ≤ 15 mm Hg, and a pulmonary vascular resistance of ≥3 WU. Approximately, 1–5% of patients with severe group 3 PH LD have a mPAP > 35–40 mm Hg [68]. 8–15% of ILD patients are categorized with group 3 PH LD with an initial mPAP > 25 mm Hg at the time of diagnosis, while 30–50% with advanced ILD and >60% with end-stage disease have similar categorization at time of discovery [68]. There has been documented research showing that a small proportion of COPD patients can present with group 3 PH LD with a mPAP > 35–40 mm Hg with a relatively preserved lung function.

Geographically, people that inhabit and reside in higher elevations and altitudes suffer from group 3 PH LD even in those patients with healthy lung physiology and development due to the pulmonary vasculature’s response to chronic and persistent low atmospheric oxygen. This physiological state mimics a similar mechanism to those with already diagnosed chronic lung diseases (i.e., COPD, ILD, OSA, CF, BPD) that causes hypoxic vasoconstriction due to low alveolar oxygen partial pressures (PAO_2_) [68]. This biochemical imbalance causes release of growth factors, and vasoconstricting hormones that will essentially increase hypertrophied vascular smooth muscle cells (VSMCs) and thereby increase vascular resistance [68].

Pulmonary vascular circulation is normally a low-pressure system with the cause of pulmonary hypertension in hypoxic LD due to a multifactorial etiology with most of the resistance being the result of the decrease in pulmonary vessel diameter as vessels branch out from the main pulmonary artery to secondary and tertiary vessels. When the heart is under stress, the cardiac output (CO) increases, the pulmonary vasculature dilates in order to compensate for the need of higher blood volumes, and under-perfused arteries are recruited to maximize this new hemodynamic state, and PAP remains approximately the same [70].

In patients with group 3 PH LD, there is a loss (or under-function) of pulmonary blood vessels that reduces the capacity to accommodate the high CO that the heart needs in order to maintain adequate tissue perfusion which causes intrapulmonary pressures to increase. Alveolar hypoxia causes contraction of the pulmonary vessels, and in the early stages this can be reversible with increased inspired oxygen concentration such as when patients on COPD utilize a home oxygen nasal cannula for supplemental oxygen needs. Chronic hypoxia causes remodeling of the pulmonary vascular system by releasing vasoconstrictors such as endothelin and serotonin, which serve as tissue growth factors that can lead to intimal hyperplasia, hypertrophy of the tunica media, and increased vascular tone [68].

Oxygen can only partially reverse the structural remodeling that has occurred intravascularly due to changes associated with group 3 PH LD. There is extensive evidence from subsequent studies that have discovered an inverse relationship between high PAP to low PAO_2_ levels [68]. Intravascularly, endothelial cells detect hypoxemia which causes depletion of nitric oxide (NO) and increases levels of endothelin-1 concentration which consequently causes contraction of VSMCs thereby increasing cellular proliferation by inhibiting anti-mitogenic factors, NO, and prostacyclins [68]. 

Subsequently, this leads to increased production of different mitogenic stimuli, like platelet-derived growth factor (PDGF) and vascular endothelial-derived growth factor (VEGF) and this cellular proliferation encouraged by these growth factors causes increased vascular tone [68]. These cellular pathways activate and inhibit molecular subunits that cause increased apoptosis of the vascular endothelium in smokers, which causes a drastic decrease in NO synthetic enzymes [68]. This ultimately creates an environment that causes a disproportionate concentration of vasodilation to vasoconstriction which causes pulmonary hypertension [68].

### 6.4. Group IV Pulmonary Hypertension Associated with Pulmonary Artery Obstructions (CTEPH)

Group 4 PH associated with pulmonary artery obstructions is often interchangeably used with group 4 chronic thromboembolic pulmonary hypertension (CTEPH) because the common etiology of group 4 PH is due to chronic thromboembolic events. According to the Pulmonary Hypertension Association (PHA), it is estimated that nearly 0.5–5% of people who develop pulmonary embolism (PE) may go on to develop CTEPH as a complication later in life. Patients that are at a higher risk for CTEPH include those that have a large PE, multiple episodes of small-to-moderate size occlusions over a long period of time, those individuals with coagulopathy and other hematological disorders that place them at higher risk for blood clots, and lastly patients that have signs of PH at the time of their PE diagnosis.

Other causes of group 4 CTEPH include malignant and non-malignant tumors, arteritis without connective tissue disease, congenital pulmonary artery stenosis, and parasites (hydatidosis) [71]. Group 4 PH results from histological fibrotic transformation of pulmonary arterial clots that complicate the microenvironment by inducing vascular remodeling [72]. The diagnosis criteria of CTEPH requires ≥ 3 months of effective anticoagulation and a mPAP > 20 mm Hg with a PAWP ≤ 15 mm Hg, and at least one (segmental) perfusion defect [72]. 

### 6.5. Group V Pulmonary Hypertension Associated with Unclear and/or Multifactorial Mechanisms

Group 5 PH associated with unclear and/or multifactorial mechanisms is caused by hematological disorders, systemic and metabolic disorders, sarcoidosis, chronic kidney disease, end-stage renal disease and renal failure, fibrosing mediastinitis, and complex congenital heart disease [71]. The hemodynamic characteristics of group 5 PH is predominantly precapillary but can also include postcapillary and combined pre- and postcapillary PH [71]. According to the PHA and Al-Qadi et al., hematological disorders like sickle cell anemia, chronic hemolytic anemia, splenectomy, and other particular blood-related disorders such as myeloproliferative disorders have been linked to placing patients at a higher risk of PH [73].

Subclassifications of group 5 PH with unclear and/or multifactorial mechanisms can include other systemic and metabolic disorders such as pulmonary Langerhans cell histiocytosis, Gaucher disease, glycogen storage disease, and neurofibromatosis [73]. Complex congenital heart diseases deriving from segmental pulmonary hypertension like isolated pulmonary artery of ductal origin, absent pulmonary artery, pulmonary atresia with ventricular septal defect and major aorto-pulmonary collateral arteries, hemi-truncus, amongst other congenital heart diseases have been investigated to cause group 5 PH [73]. Single ventricle congenital heart disease like operated Scimitar syndrome—a rare congenital cardiac defect where the right lung veins drain abnormally, often causing an outflow into the inferior vena cava and coinciding with a hypoplastic right lung or pulmonary artery and single ventricle physiology—can cause group 5 PH [73].

## 7. Diagnostic Modalities and Imaging Studies

### 7.1. Echocardiography

**Figure 2:**
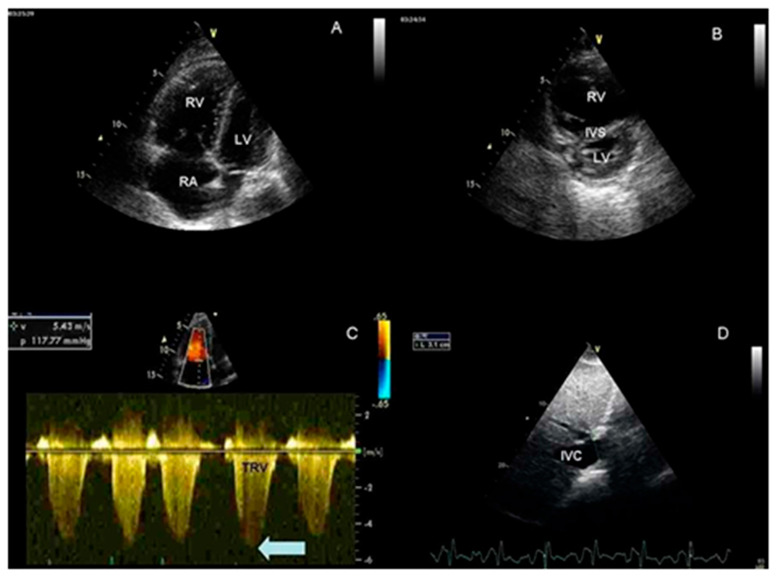
Echocardiography of a patient with idiopathic pulmonary arterial hypertension. (**A**,**B**) Apical 4-chamber and parasternal short-axis views, showing severe RV enlargement and pathologic migration of the IVS toward the LV. (**C**) Doppler tracing of TR, showing severe PH (TRV > 5 m/sec) (arrow). (**D**) Dilated IVC from the subcostal view. **RA** = right atrium; **RV** = right ventricle; **LV** = left ventricle; **IVS** = interventricular septum; **TR** = tricuspid regurgitation; **PH** = pulmonary hypertension; **TRV** = tricuspid regurgitation peak velocity; **IVC** = inferior vena cava. Reprinted/adapted with permission from Ref. [79].

There are many imaging modalities that can aid in diagnosis of pulmonary hypertension. Imaging of the heart is essential; this is due to the intimate connection between the pulmonary artery and the heart chambers. Echocardiogram (Figure 2) and right heart catheterization are two imaging techniques that are extremely important since right ventricular failure is the best predictor and most common cause of death in patients with pulmonary arterial hypertension [74]. Echocardiogram findings in patients with pulmonary hypertension include right ventricle and right atrium dilation, right ventricle hypokinesis, septal flattening or bowing toward the left ventricle, tricuspid regurgitation, pulmonary insufficiency, and mid-systolic closure of the pulmonary valve [75]. The right ventricle is typically smaller than the left ventricle, on echocardiogram if you see a much larger right ventricle and right atrium, pulmonary hypertension should be high on the differential. This is because the right side of the heart has to pump against an increased pressure in the pulmonary artery, eventually leading to dilation. 

The tricuspid annular plane systolic excursion/systolic pulmonary artery pressure (TAPSE/sPAP) ratio can be utilized via the incorporation of echocardiographic imaging as a map to assess pulmonary hypertension hemodynamics and prognostic severity. This metric will gauge right ventricular-pulmonary arterial (RV-PA) coupling where lower ratios (i.e., <0.55 mm/mm Hg) indicate increased risk of mortality [76]. Further clinical research on the hemodynamics of PAH has shown that RV-PA coupling assessed by TAPSE/sPAP ratios has led to more improved risk assessment scores as an indication for prognostic factors in all metrics except in the lowest or most advanced stages of the disease [77]. Systolic pulmonary artery pressure (sPAP) and the acceleration time of pulmonary outflow (PAAT) are variables that can be incorporated along with echocardiographic imaging studies to calculate PVR, and pulmonary arterial ventricle interaction in patients with PAH [78]. The right ventricular arterial coupling (RV-AC) has also been utilized as another metric to gauge pulmonary vascular load and hemodynamic pressure changes [78].

### 7.2. Right Heart Catheterization

Right heart catheterization (RHC) is the gold standard for diagnosing pulmonary hypertension. It allows the clinician to directly measure the pressures in the pulmonary artery [80]. The primary downside with right heart catheterization is the fact that it is invasive and can have complications including perforation, arrhythmias and bleeding. RHC is vital to accurately and precisely diagnose PH as traditional hemodynamic studies may be insufficient to identify early stages of the disease, and its etiology, which will thereby lead to misinterpretation of possible treatment decisions and therapeutic modalities [80].

There is also a barrage of complementary studies and maneuvers that can be accompanied with RHC in order to test the pulmonary vasculature with the sole outcome of diagnosing PH. Maneuvers such as established tests including vasodilator testing, exercise, fluid administration and other promising tests requiring further research like passive leg raising, esophageal balloon and temporary arterio-venous dialysis access occlusion and dobutamine infusion are viable options in order to challenge the pulmonary vasculature and influence hemodynamic circulation in hopes of diagnosing pulmonary hypertension [80]. 

For example, dobutamine infusion utilized in concert with RHC can be beneficial in determining right ventricular contractile reserve but comes at a great cost. The risks of dobutamine infusion in a patient that also has a concomitant hypertrophic cardiomyopathy with LV outflow tract obstruction or arrhythmias is that it may greatly exacerbate this complication [80]. Another example would be the usage of esophageal balloons in the diagnosis of PH. Esophageal balloons can accurately determine pulmonary hemodynamics in obese patients or other subjects that already are predisposed or have a baseline elevation in pleural pressures [80].

### 7.3. Computed Tomography Angiography and Computed Tomography Pulmonary Angiography

**Figure 3:**
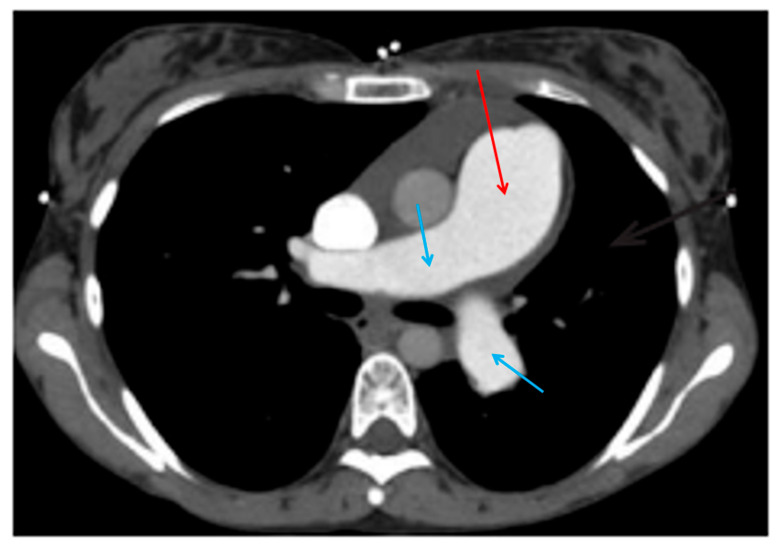
Computed tomography angiography highlighting pulmonary artery dilatation (red arrow) and dilatation and vessel widening of downstream vasculature (blue arrows) due to increased pulmonary arterial pressures in the pathological state of pulmonary hypertension. Reprinted/adapted with permission from Ref. [82].

Computed tomography (CT) angiography (Figure 3) is another helpful imaging study, specifically CT coronary angiography and CT pulmonary angiography. In patients with angina and pulmonary arterial hypertension, CT coronary angiography is great at diagnosing left main coronary artery (LMCA) compression. The LMCA can get compressed in pulmonary hypertension due to pulmonary artery dilation, which pushes on the LMCA. The compression leads to obstruction of blood flow through the artery and presents with anginal symptoms [81]. The prevalence of left main coronary artery compression in patients with pulmonary hypertension and angina is high.

A study by Galiè et al. included 765 patients with pulmonary hypertension, 121 had angina or angina-like symptoms. A total of 94 patients had abnormal CT coronary angiography based on the relationship between the pulmonary artery and the left main coronary artery and underwent selective coronary angiography. Left main coronary artery stenosis ≥ 50% was detected in 48 of the 94 patients. A total of 45 patients underwent PCI with stenting, and 41 of these patients had sustained angina symptom relief. The 3 other patients had surgical pulmonary artery reduction plasty. Nine months after PCI, 5 patients had left main coronary artery restenosis and PCI was successfully repeated. The best predictor of left main coronary artery stenosis ≥ 50% was a pulmonary artery diameter ≥ 40 mm [81]. 

The results from Galiè et al. suggest that CT coronary angiography is indicated in patients with pulmonary hypertension and angina. PCI was seen to improve symptoms and long-term outcomes in patients [81]. CT pulmonary angiography on the other hand has been shown to be a great diagnostic tool for pulmonary hypertension because it can accurately measure the diameter of the pulmonary artery. The Framingham study assigned 29 mm and 27 mm as representing the upper limits of normal pulmonary artery diameter for male and female patients respectively. Past research using pulmonary artery diameter to diagnose pulmonary hypertension has shown that a diameter greater than 29 mm had a sensitivity of 75% and a specificity of 89% for the presence of pulmonary hypertension [83]. However, it is important to note that pulmonary artery diameter can be increased in the absence of pulmonary hypertension, and thus, increasing diameter of pulmonary artery does not strongly correlate with increasing mean pulmonary artery pressures [83].

### 7.4. Lung Ventilation and Perfusion Scan

**Figure 4:**
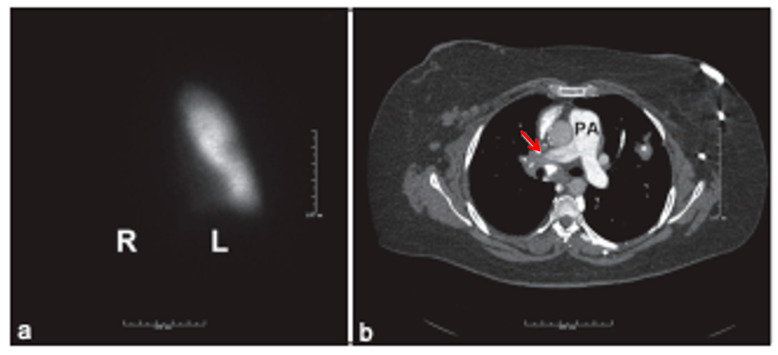
Ventilation perfusion scan and computed tomography angiography of a patient with pulmonary hypertension in the setting of fibrosing mediastinitis and absent flow to the right lung. (**a**) The lack of perfusion on the right lung. (**b**) An obstruction of the right main pulmonary artery (*red arrow*) due to fibrosing mediastinitis. **R** = right lung; **L** = left lung; **PA** = pulmonary artery. Reprinted/adapted with permission from Ref. [84].

Lung ventilation (V) and perfusion (Q) scan (V/Q scan) (Figure 4) is another helpful tool because it can exclude chronic thromboembolic pulmonary hypertension (CTEPH) with a negative predictive value of 98%. Chronic thromboembolism as the cause of pulmonary hypertension often goes undetected or underrecognized because previous pulmonary embolism is not always apparent. Guidelines recommend a V/Q scan as the initial test when there is a high suspicion of CTEPH; if the V/Q scan is positive, then CT angiography is an appropriate next step to identify changes in vascular morphology such as intimal irregularities, webs, and luminal narrowing [2]. A V/Q scan is an effective screening tool in CTEPH as a negative V/Q scan virtually rules out CTEPH in patients with concerns of group 4 pulmonary hypertension [84].

A normal- or low-probability V/Q scan effectively excludes CTEPH when compared to CT pulmonary angiography with the former having a sensitivity of 90–100% and a specificity of 94–100%, while the latter has a wide range of sensitivity between 50 and 98% with similar specificity [84]. Even though it has a high sensitivity, lung V/Q scans for detecting not only CTEPH but also for detecting distal forms of the disease are underutilized, and thus high-resolution CT imaging studies of the lungs are often used instead due to availability [84]. V/Q studies in CTEPH reveal wedge-shaped perfusion defects with normal ventilation, but in complete absence of perfusion to one lung, imaging results can be indicative of conditions such as malignancy, vasculitis, and fibrosing mediastinitis [84].

However, careful analysis and accurate result interpretation must be conducted on routine unmatched perfusion studies and their respective defects which can manifest in the diagnosis of other pulmonary vascular diseases, such as pulmonary veno-occlusive disease (PVOD) or pulmonary capillary hemangiomatosis (PCH) which can cause devastating complications such as pulmonary edema which can cause or exacerbate an already hypoxic state should these patients be treated with pulmonary vasodilator agents [84]. The limitations of V/Q scans include the potential for underestimating the extent of central vascular obstruction and its inability to differentiate between similarly presenting diseases like PVOD, PCH, or fibrosing mediastinitis [84]. Besides the ease of use, high sensitivity and specificity, V/Q scans have an advantage over CT pulmonary angiography in that it does not utilize iodine contrast and has less radiation exposure with an effective dose for thoracic imaging of 0.6–3 mSv compared to 8–20 mSv for a multi-detector CT scan [84].

### 7.5. Cardiac Magnetic Resonance Imaging

**Figure 5:**
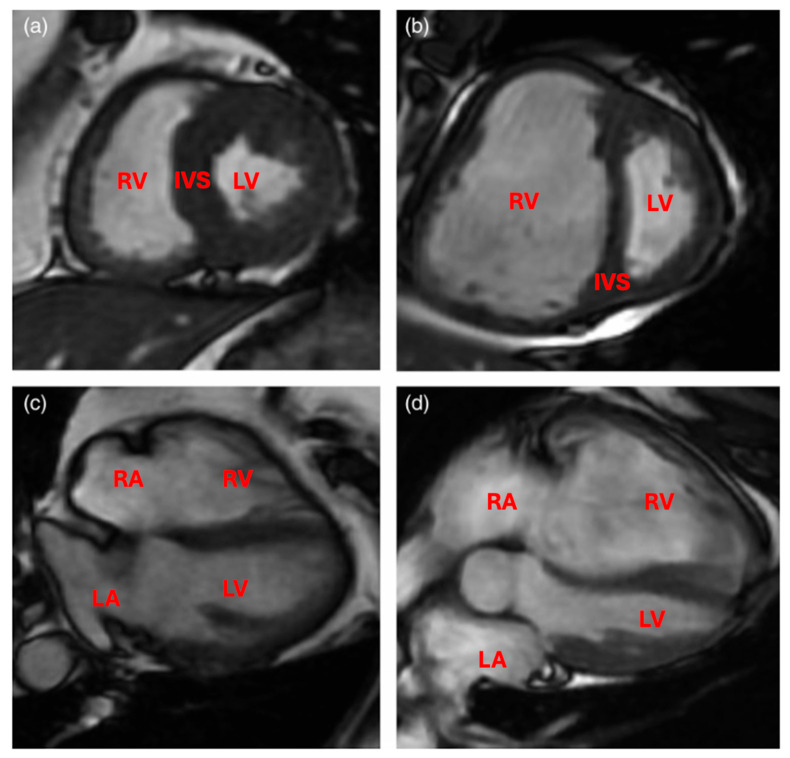
Cardiac MRI study of a patient with and a patient without pulmonary hypertension (PH). (**a**,**c**) Short-axis and 4-chamber view of a patient without PH; (**b**,**d**) Short-axis and 4-chamber view of a patient with PH. In patients with PH, elevated RV pressure causes RV hypertrophy and leads to increased septal angle deformity. **RV** = right ventricle; **IVS** = interventricular septum; **LV** = left ventricle; **RA** = right atrium; **LA** = left atrium; **MRI** = magnetic resonance imaging. Reprinted/adapted with permission from Ref. [86].

Lastly, MRI is an underutilized tool in the workup of pulmonary hypertension. Cardiac magnetic resonance imaging (CMRI) specifically can look at the structure of the heart and pulmonary vasculature. The utility of cardiac MRI is that not only can it aid in diagnosis, but it can also help determine the prognosis in patients with pulmonary hypertension (Figure 5); right ventricular ejection fraction measured by MRI can help calculate the 1-year mortality rate [85]. CMRI can overcome some of the limitations that are exposed by utilizing transthoracic echocardiography (TTE) with respect to the assessment of the right ventricle [84]. CMRI assesses RV volumes and function, and it gives clear definition of the interface contour between the blood–myocardium interface and analytical expansion of the RV volume, mass, and function [84].

The benefits of CMRI include the lack of ionizing radiation exposure but at a cost of it being an expensive imaging modality, not being widely available and also requiring operator expertise [84]. CMRI evaluates pulmonary artery distensibility and can correlate this to vasodilator therapy response, with a sensitivity of 100% and specificity of 56% with proximal pulmonary artery stiffness having been shown to predict mortality in patients with PH [84]. Serial CMRIs have been shown to be reliable and accurate in monitoring treatment response and prognosis of PH. Another modern assessment to better diagnose and gauge the impact of pulmonary hypertension on cardiac hemodynamics includes the discussion of right ventricle–pulmonary artery (RV-PA) coupling.

RV-PA coupling is the intricate linkage amongst the right ventricle (RV) contractility and pulmonary artery (PA) afterload, which acts as a crucial indicator for energy transfer efficiency by modulating the optimal balance of RV contractility, pulmonary vascular resistance, and compliance to sustain RV-PA coupling [85]. A study conducted to investigate RV overload and the clinical consequences of PAH showed that coupling parameters of RV-PA were linked with glucose metabolism and molecular uptake in cardiomyocytes when assessed with CMR and 18-Fluorodeoxyglucose (FDG) positron emission tomography (PET) [87]. The investigation concluded that the utilization of PET and CMR alluded to the confirmation amongst the association of RV-arterial uncoupling with higher metabolic demand which can lead to PAH deterioration [87]. Another utility of RV-PA coupling is the link it has with multi-beat pressure-volume loops and can predict transplant-free survival in patients with PAH [88].

### 7.6. Positron Emission Tomography

**Figure 6:**
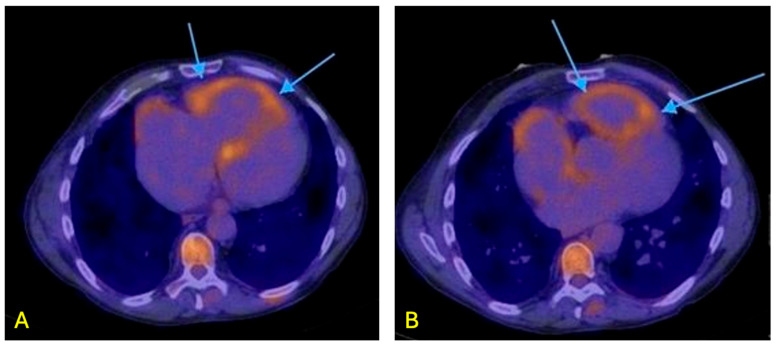
Cardiac PET with ^18^F-fluorodeoxyglucose (FDG) tracer. (**A**,**B**) Arrows showing extensive FDG uptake involving the entire right ventricle wall, the right atrium, and the interventricular septum with additional absence of left ventricle free wall FDG uptake. Reprinted/adapted with permission from Ref. [91].

Positron emission tomography (PET) imaging, particularly utilized with tracers like FDG offer clinicians a non-invasive method to detect and monitor PAH hemodynamics, metabolic changes, and tissue remodeling. In a normal non-pathologic state, approximately 95% of metabolic energy utilized by the myocardium comes from mitochondrial oxidative phosphorylation, with most coming from fatty acid metabolism, a lesser extent from carbohydrate metabolism and the remainder from glycolysis [89]. Increased pulmonary artery pressures are associated with higher ^18^F-fluorodeoxyglucose (FDG) uptake (Figure 6) in the right ventricle while assessing these findings with the utilization of PET imaging studies [89]. This preferential ‘metabolic shift’ which occurs in cardiomyocytes is due to pressure overload in the RV, causes high uptake of FDG which correlates to PAH progression and worsening outcomes and deterioration [89]. Some studies have even suggested the use of late gadolinium enhancement in myocardial tissue as a prognostic marker in patients with PH [90].

All the imaging studies mentioned above have a role in the diagnosis or subsequent management of pulmonary hypertension including their advantages and disadvantages (Table 3). 

## 8. Pathophysiology

The etiology of pulmonary arterial hypertension includes heritable PAH, which includes the gene *BMPR2* in the vast majority of genetic related cases, as well as *ALK1*, *ENG*, *SMAD9*, *CAV1*, and *KCNK3*; drug-and-toxin-induced PAH, with drugs such as fenluramine, amphetamines, cocaine and SSRIs; and PAH due to associated disease, with diseases including connective tissue disease, HIV, congenital heart disease and schistosomiasis [92,93,94,95]. Regardless of the etiology of pulmonary arterial hypertension, the pathophysiology converges on three main points: fibromuscular intimal thickening, vascular smooth muscle cell hypertrophy, and endothelial cell damage. These three pathological changes are the primary contributors to the decreased cross-sectional area in the pulmonary vessels and increased vascular stiffness [96]. 

The cells that mediate the fibromuscular intimal thickening and smooth muscle cell hypertrophy are the pulmonary artery endothelial cells, pulmonary artery smooth muscle cells, and pulmonary artery fibroblasts [71]. Cell signaling pathways can get disrupted leading to unchecked proliferation of these three cell types; unchecked proliferation and hypertrophy of the vasculature make the vessels stiff and noncompliant, leading to increases in the pulmonary arterial pressures. Bone morphogenetic receptor protein 2 (BMPR2) is the primary genetic component involved in pulmonary arterial hypertension. It is a homeostasis factor in the pulmonary endothelial cells and smooth muscle cells; when there is a loss of function mutation in *BMPR2* gene, there is abnormal proliferation of the endothelial and smooth muscle cells, which strongly contributes to the increase in vascular resistance since the vessels become noncompliant and hypertrophied.

Direct damage to the pulmonary artery endothelial cells themselves is also thought to be a strong contributor to pulmonary arterial hypertension. Different drugs and toxins can damage the endothelium through an inflammatory response, while associated connective tissue disease predisposes people to endothelial dysfunction. The primary ways endothelial cells get damaged is from inflammation, hypoxia or shear stress; the endothelium becomes damaged to the point where the cells are not able to produce enough nitric oxide or prostacyclin, and end up producing increased amounts of thromboxane and endothelin-1 [97]. Nitric oxide and prostacyclin are vasodilating agents that help the vessels comply with increases in pressure or vascular wall stress, while thromboxane and endothelin-1 are vasoconstrictors [98]. 

The imbalance between the vasodilators and vasoconstrictors leads to the increased pressures in the pulmonary vasculature. Endothelial damage not only leads to this imbalance, but it is also one of the main triggers of remodeling in vessels. Damage from stress (shear, inflammation, hypoxia) causes apoptosis, the endothelial cells start the process of apoptosis, and this leads to a decrease in pulmonary vessels. The remaining endothelial cells are resistant to apoptosis and begin to hyper-proliferate into abnormal and dysfunctional plexiform lesions. Eventually, the cells become senescent and render the pulmonary artery hypertension essentially irreversible. The result of remodeling of the endothelium is muscularization and occlusion of the lumen of pulmonary arteries through the formation of vascular lesions [97]. 

## 9. Complications

**Figure 7:**
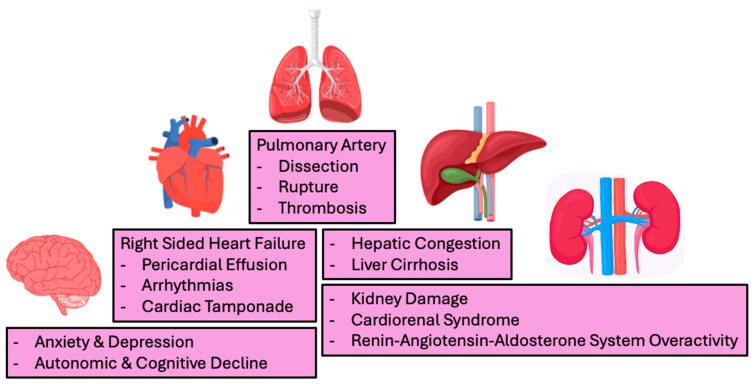
End-organ system dysfunction and complications caused by pulmonary arterial hypertension.

The complications of pulmonary arterial hypertension are broad (Figure 7) and span many organ systems. These organ systems include the brain, lungs, heart, kidneys and liver. Many of the complications of pulmonary hypertension are connected through the development of right-sided heart failure. Right-sided heart failure predisposes the patient to arrhythmias, pericardial effusion, cardiac tamponade, liver cirrhosis, and kidney damage. Complications independent of right-sided heart failure include pulmonary artery dissection or rupture, thrombosis, and extrinsic compression of surrounding structure via pulmonary artery dilatation [99].

### 9.1. Brain

Pulmonary arterial hypertension takes a huge toll on brain health and as such patients often have depression, anxiety, autonomic and cognitive decline which could suggest that the parts of the brain that control these functions are being affected. Roy et al. looked at T1 and T2 brain MRI images from 9 PAH and 19 healthy subjects. Gray matter volume was calculated and used to compare differences between gray matter volume in PAH patients and the controls [100]. Roy et al. found that people with PAH had significantly decreased gray matter volumes. The regions of decreased gray matter included the hippocampus, insula, cerebellum, para-hippocampus, temporal, frontal, and occipital gyri, cingulate, amygdala, and the thalamus [100]. They concluded that PAH patients showed significant gray matter injury and brain tissue alterations in sites that help regulate cognition, autonomic function, and mood, giving a structural basis to the functional deficits seen in people with PAH [100]. 

### 9.2. Lungs

Complications of PAH that are intrinsic to the lung vasculature include dissection or rupture, thrombosis, and post-operative respiratory failure. Pulmonary artery dissection is a rare but very fatal complication of pulmonary hypertension. The increased pressure predisposes the endothelium to damage, allowing for blood to dissect through the intima into the media. This condition typically ruptures leading to sudden death due to cardiogenic shock and cardiac tamponade, it is so fatal that the vast majority of cases are confirmed post-mortem [101]. Thrombosis is not only a complication of pulmonary hypertension, but is also a cause. Pulmonary hypertension affects the flow and predisposes the endothelium to damage, which makes platelets aggregate and creates a prothrombotic environment where thrombi are more likely to form [102]. 

Pulmonary hypertension increases the post-operative mortality and morbidity in patients undergoing surgery. They have increased risk for pulmonary complications and even respiratory failure shortly after undergoing surgery [103]. A study by Kruthiventi et al. looked at patients with pulmonary hypertension who underwent surgery with general anesthesia from 2010 to 2017, and monitored them for post-operative pulmonary complications. These pulmonary complications included hypoventilation, hypoxemia, apnea and pain-sedation mismatch. From a total of 128 patients with pulmonary hypertension who had undergone 197 surgical procedures, 20 post-operative pulmonary complications were noted, which gave a complication rate of 10.2% in patients with pulmonary hypertension. This suggests that patients with pulmonary hypertension should be closely monitored in the immediate post-operative care period [103].

### 9.3. Heart

Right-sided heart failure as a result of pulmonary hypertension leads to the vast majority of complications and contributes to the majority of deaths in patients with pulmonary hypertension. Right-sided heart failure develops due to the increased resistance in the pulmonary vasculature, the right ventricle will struggle to pump against increasing pressure and eventually fail after maladaptive fibrosis, hypertrophy and dilatation occurs [104]. The maladaptive fibrosis, dilatation and hypertrophy affect the electrical system throughout the right ventricle, predisposing to arrhythmias. Arrhythmias are important contributors to morbidity and mortality in patients with pulmonary hypertension [105]. It is theorized that in people with right ventricle dysfunction, voltage-gated potassium channels are downregulated leading to prolonged QTc waves on EKG, and thus increasing the chance of developing a life-threatening cardiac arrhythmia; however, this has only been studied in rats, not humans [106]. 

Regardless, a study conducted on 201 patients with pulmonary hypertension demonstrated that the QTc significantly increased in patients with severe pulmonary hypertension when compared to mild or moderate, even if the cause of the QTc prolongation has not been fully studied [107]. In addition to arrhythmias, right-sided heart failure can cause the development of pericardial effusions through increased hydrostatic pressure and impaired fluid resorption. Studies found a pericardial effusion prevalence of 29%, and over a 2-year follow-up period, they found an incidence of 44.1% [108]. Pericardial effusion development can predispose individuals to cardiac tamponade. But cardiac tamponade in PH has an atypical presentation because right atrial and ventricular diastolic collapse, pulsus paradoxus, and hypotension are usually absent [108]. The elevated right-sided pressures are higher than the pericardial pressure, preventing the right ventricular diastolic collapse usually seen in cardiac tamponade [108]. However, classic cardiac tamponade can develop from pulmonary artery rupture, as described previously. This occurs due to rupture of the intrapericardial portion of the pulmonary artery, ultimately leading to blood accumulation in the pericardial sac and the typical compressive symptoms. 

Increases in pulmonary arterial pressure predispose the vessel to dilatation; if the dilatation is severe enough, it can compress surrounding structures such as the left main coronary artery, the left recurrent laryngeal nerve, and the tracheobronchial tree [109]. The compression of the left main coronary artery can present as angina; compression of the left recurrent laryngeal nerve can present as hoarseness; and compression of the tracheobronchial tree can present with dyspnea. Of these, compression of the left main coronary artery poses the greatest risk in terms of sudden death. There is a strong correlation between pulmonary artery size and compression of left main coronary artery, since pulmonary artery hypertension is the most common cause of increased pulmonary artery diameter, left main coronary artery compression must be considered [81]. 

### 9.4. Kidneys

Renal complications occur in patients with pulmonary hypertension due to the development of right-sided heart failure and subsequent cardiorenal syndrome. Studies have found that a decrease in cardiac output and increased central venous pressures are the two critical components when looking at why renal complications arise. Patients with right-sided heart failure have decreased cardiac output; this leads to fluid overload as the heart cannot adequately pump blood arriving through the vena cava. The venous pressures increase and pressures are transmitted back to the renal efferent arterioles, resulting in a decline in glomerular filtration pressure and eventual renal injury [110]. Activation of the renin–angiotensin–aldosterone system (RAAS) and the sympathetic nervous system is what links heart and kidney damage in cardiorenal syndrome. Continued overactivity of RAAS and the sympathetic nervous system increases oxidative stress, inflammation, and fibrotic extracellular matrix remodeling in the heart, increasing the dysfunction [110]. The worsening heart failure damages the kidneys through renal congestion from fluid overload, and decreased renal perfusion. 

### 9.5. Liver

Liver disease and cirrhosis can occur as a consequence of pulmonary arterial hypertension due to increased pressures in the venous system from right-sided heart failure. The elevated pressure in the vena cava is transmitted through the hepatic veins to the liver; this congestion leads to damage of hepatocytes [111]. If left untreated, the damaged and dying hepatocytes from hepatic congestion release inflammatory signals, which activate hepatic stellate cells to produce excessive fibrous tissue, ultimately progressing to cirrhosis [112]. Since cirrhosis has a high morbidity and mortality, it should be closely monitored for as a complication of pulmonary hypertension [7,113]. 

## 10. Management and Treatment

Pulmonary hypertension (PH) is a chronic, progressive syndrome for which a definitive cure is uncommon outside select etiologies, such as operable chronic thromboembolic pulmonary hypertension. Management strategies including lifestyle modifications and strict adherence to reductions in maladaptive behaviors that can increase the incidental risk of PH or worsen its complications is of utmost importance. Other management practices are focused on symptom relief, prevention and treatment of right ventricular (RV) failure, reduction in clinical worsening, and improvement in survival. PH is not treated as a single disease entity; therapeutic decisions are determined by the World Health Organization (WHO) group, hemodynamic phenotype, disease severity, and longitudinal risk status. Treatment of PH lies in the design of targeted biochemical molecular receptors and structures including the utilization of conventional medical therapies which consists of traditional pharmacological modalities. Lastly, under dire and more serious situations and clinical indications, surgical interventions including lung transplant is another viable option, albeit coming with great risks including several post-operative complications. Contemporary guidelines emphasize structured risk assessment at diagnosis and at regular follow-up, with escalation of therapy until low-risk status is achieved or advanced therapies are pursued [2,11].

### 10.1. Lifestyle Modifications, Supportive and Symptom-Directed Therapies

Lifestyle modifications including supplemental oxygen is indicated for patients with resting or exertional hypoxemia, as chronic alveolar hypoxia promotes pulmonary vasoconstriction and vascular remodeling. Long-term oxygen therapy (LTOT) may stabilize pulmonary artery pressures and improve symptoms, particularly in PH associated with chronic lung disease [114]. Supervised exercise training and pulmonary rehabilitation in clinically stable patients improve functional capacity and quality of life and are recommended as part of comprehensive care [2]. Vaccination against influenza and pneumococcus reduces respiratory infection–related decompensation. Smoking cessation and avoidance of environmental or occupational pulmonary toxins are mandatory, and anorexigenic agents linked to PAH such as aminorex, fenfluramine, dexfenfluramine, and benfluorex must be strictly avoided. Stimulants like methamphetamine, cocaine, and phenylpropanolamine (PPA) which have implications of causing direct vascular toxicity should also be discouraged and prohibited.

Comorbid conditions that worsen pulmonary vascular load or RV function—including obstructive sleep apnea, anemia and iron deficiency, thyroid disease, and arrhythmias—should be aggressively treated [2]. In PAH, pregnancy is associated with high maternal mortality and is generally discouraged, requiring explicit counseling and coordinated specialty management [2]. Psychosocial support and palliative care should be integrated early in patients with persistent symptoms, progressive RV failure, or recurrent hospitalizations. Supportive and symptom-directed therapy is foundational in all forms of PH and is used in parallel with disease-targeted treatment. RV failure commonly leads to systemic congestion, which is managed with loop diuretics such as furosemide or bumetanide, with thiazide-type diuretics added in refractory cases. Judicious titration is required to relieve edema, ascites, and hepatic congestion while avoiding excessive preload reduction, hypotension, or renal dysfunction [2].

### 10.2. Group I Pulmonary Arterial Hypertension: Algorithm-Based Therapy and Escalation

Group 1 PH includes idiopathic etiologies, heritable and genetic linkage; PAH induced by medications, drugs, or toxins or associated with connective tissue disease; HIV infections, portal hypertension, congenital heart disease, schistosomiasis, pulmonary veno-occlusive disease, pulmonary capillary hemangiomatosis; and persistent PH of the newborn [71]. PAH is the PH subtype with the most robust evidence base for targeted therapy and is managed using a structured, risk-driven treatment algorithm. Risk stratification in PAH is performed using validated multiparametric tools that estimate short- and intermediate-term mortality and directly inform therapeutic intensity. In contemporary practice, commonly used models include the ESC/ERS risk stratification framework as well as registry-derived tools such as the REVEAL 2.0 and REVEAL Lite 2 risk scores. The REVEAL risk score integrates clinical variables, biomarkers, imaging findings, and—in its full version—hemodynamic parameters to generate a numerical estimate of mortality risk and is frequently used in clinical practice and trials to guide escalation to combination therapy, initiation of parenteral prostacyclin, and referral for lung transplantation [115,116].

Current guidelines recommend initial risk stratification followed by upfront combination therapy in most patients without major cardiopulmonary comorbidities [2]. In contemporary practice, the standard initial regimen for low- and intermediate-risk patients consists of dual oral therapy with an endothelin receptor antagonist (ERA) (e.g., Bosentan, Ambrisentan, and Macitentan) and a phosphodiesterase-5 inhibitor (PDE-5i) (e.g., Sildenafil and Tadalafil). Commonly used combinations include ambrisentan plus tadalafil or macitentan plus tadalafil or sildenafil. This strategy targets both endothelin-mediated vasoconstriction and nitric oxide–cGMP signaling and has been shown to improve exercise capacity and reduce clinical failure compared with monotherapy, as demonstrated in the AMBITION trial, and is endorsed by international guidelines [2,65]. 

Other medications that target soluble guanylate cyclase, prostacyclin, prostacyclin analogues, and calcium channel blockers (CCB) can also be utilized for the management of group 1 pulmonary arterial hypertension [71]. Prostaglandins such as epoprostenol, iloprost, selexipag, and treprostinil along with soluble guanylate cyclase (sGC) stimulators like riociguat are effective therapies which will be discussed later in great detail. CCB like nifedipine, diltiazem, nicardipine and amlodipine can also be effective in helping relax the arteries of the lungs and thereby reducing pulmonary blood pressures.

PDE-5 degrades cyclic guanosine monophosphate (cGMP), which is the product of NO production and directly acts on PASMCs to induce vasodilation; thus, PDE-5 inhibitors like Sildenafil and Tadalafil help stabilize cGMP levels and assist in supporting a cellular environment leading to vasodilatory effects [114]. According to the National Health Service (NHS), which is the United Kingdom’s publicly funded healthcare system, prostacyclins, which are derivatives of prostaglandins, are viable treatment options for group 1 PH. 

Patients with group 1 PH are reassessed at regular intervals, typically every 3–6 months, using functional class, exercise capacity, biomarkers such as NT-proBNP, echocardiography, and hemodynamics when indicated. If low-risk status is not achieved or maintained, therapy is escalated rather than observed, consistent with guideline-directed treat-to-low-risk strategies supported by ESC/ERS guidance and REVEAL-based outcome data [2,116]. Escalation most commonly involves incorporation of the prostacyclin pathway, resulting in triple therapy. This may include addition of oral selexipag to background ERA and PDE-5i therapy, supported by outcome-driven data from the GRIPHON trial, which demonstrated reduced morbidity and mortality across a broad PAH population, as well as guideline recommendations supporting prostacyclin-pathway escalation [2,117]. Inhaled prostacyclin formulations such as inhaled treprostinil or iloprost may also be used in selected patients.

Patients with high-risk features at diagnosis or during follow-up—including severe RV dysfunction, markedly elevated right atrial pressure, low cardiac index, and advanced functional class—should receive early parenteral prostacyclin therapy, most commonly intravenous epoprostenol or intravenous/subcutaneous treprostinil, in combination with ERA and PDE-5i or soluble guanylate cyclase-stimulating therapy. Parenteral prostacyclins remain the most potent disease-targeted therapies in PAH and are strongly recommended in high-risk disease by contemporary guidelines [2]. Calcium channel blockers are reserved exclusively for patients who demonstrate acute vasoreactivity during right heart catheterization.

**Figure 8:**
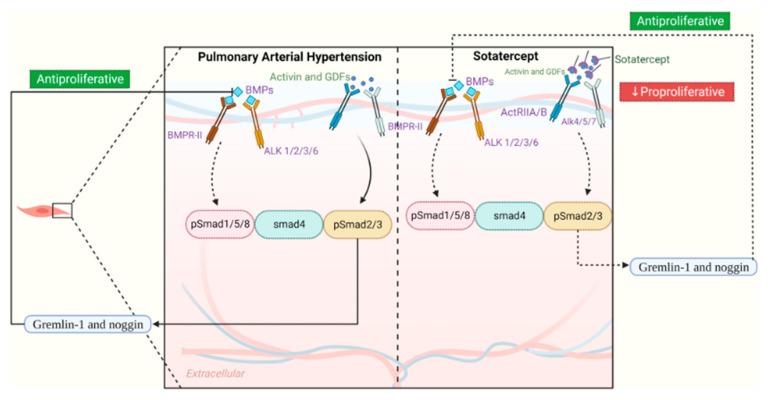
The mechanism of action of sotatercept involves the binding and inhibition of Pro-proliferative activin-class ligands (Activin A, GDFs 8 and 11) which intrinsically promote cell growth and myocyte proliferation. The inhibition of activin and GDFs by sotatercept leads to downstream prevention and deactivation of ActRIIA/B and the pSmad2/3 pathway. This in turn causes downregulation of endogenous BMP antagonists, Gremlin-1 and noggin. This causes a reflex which allows the support of growth-inhibiting pathways via the anti-proliferative BMPs to dominate causing activation of BMPR-II thereby reversing vascular remodeling and decreasing elevated pulmonary arterial pressure. Reprinted/adapted with permission from Ref. [120].

A major recent advance in PAH management is the introduction of sotatercept, a first-in-class activin-signaling inhibitor targeting pulmonary vascular remodeling. Sotatercept was approved by the U.S. Food and Drug Administration in 2024 for adults with WHO Group 1 PAH as an add-on to background therapy to improve exercise capacity and reduce clinical worsening. In the phase 3 STELLAR trial, sotatercept significantly improved pulmonary vascular resistance, six-minute walk distance, and functional class when added to establish PAH regimens, including dual and triple therapy [118]. These findings were subsequently reflected in regulatory approval and clinical guidance [119]. Unlike traditional vasodilators, sotatercept directly addresses maladaptive vascular proliferation (Figure 8), reinforcing the importance of early diagnosis before irreversible remodeling occurs.

Sotatercept is a fusion protein that is inherently designed to “balance the scales” between pro-proliferative or pathological mechanisms and anti-proliferative or protective mechanisms via the dampening of the transforming growth factor-beta (TGF-β) superfamily members including Activin A, GDF8, and GDF11 and their inactivation of the BMP pathway [118]. Sotatercept binds and sequesters free, circulating ligands that are abundantly expressed in patients with PAH and inhibits them from binding to their target cellular receptors. The molecular makeup and structural components of sotatercept involves a recombinant fusion protein with an extracellular domain of activin receptor type IIA (ActRIIA) connected to the Fc domain of an IgG1 [118]. Ligands such as Activin A, Activin B, GDF8, and GDF11 falsely bind to sotatercept’s decoy ActRIIA domain and this prevents the ligands intended effects of activating downstream pro-proliferative signaling cascade [118].

Sotatercept also has influence on the inhibition of the pro-proliferative SMAD pathway via the prevention of ligands Activin A and GDFs (8/11) which act on pulmonary vascular smooth muscle cells and endothelial cells that facilitate PAH disease progression [118]. When ligands Activin A and GDFs (8/11) go uninhibited they activate ALK4, ALK5, or ALK7 receptor complexes which in turn phosphorylates Smad2 and Smad3 turning them into pSMAD2/3 [118]. The deleterious impact of pSmad2/3 activation encourages and fosters an environment where further proliferation, vascular remodeling, and decreased apoptosis in the pulmonary vasculature goes unchecked leading to severe lumen narrowing [118]. Sotatercept traps the initial ligands thereby preventing ALK4/5/7 activation and inhibiting pSMAD2/3 formation [118].

Activating the anti-proliferative pathway in driven by sotatercept’s restoration of homeostasis or the concept of “balancing the scales” where pathological remodeling or the pulmonary vasculature is reversed by allowing the endogenous BMP pathway to regain dominance [118]. By inhibiting the pSMAD2/3 complex to form, sotatercept is essentially supporting the BMPR-II receptor which was previously overwhelmed to thereby activate and signal via the pSMAD1/5/8 pathway [118]. The lack of pSMAD2/3 activity allows further inhibition of endogenous antagonists like Gremlin-1 and noggin protein units to not bind and inhibit BMP ligands like BMP2/4/7 [118]. In conclusion, a decrease in Gremlin-1/noggin units allows BMPR-II/pSMAD1/5/8 signaling to be restored and unrestricted which thereby promotes and facilitates apoptosis of pathologic cells, and inhibition of proliferation which ultimately leads to reduction in vascular medial hypertrophy and improved hemodynamics [118].

Patients with progressive PAH despite optimized medical therapy should be managed at specialized PH centers, where escalation to parenteral prostacyclin, addition of sotatercept when appropriate, enrollment in clinical trials, and early transplant referral can be coordinated [2]. Invasive procedures for the treatment of group 1 PH can include atrial septostomy (AS) which involves the creation of a conduit between both the atria in order to facilitate unloading of the right-sided cardiac chambers has also shown to improve pulmonary hemodynamics, but its 30-day mortality can be as high as 25% [121]. AS may be considered in highly selected patients with refractory RV failure as a bridge to lung transplantation similar to how an LV assist device (LVAD) is considered a viable bridge to heart transplantation [121,122].

Other medications used for the treatment of group 1 PAH include tacrolimus which is a calcineurin inhibitor and a primary agent in inducing immunosuppression in transplant patients. Tacrolimus acts on the BMPR2 signaling pathway and inhibits the interaction between the molecular receptor and its repressor, FKBP12, thus enhancing the BMP pathway in the context of BMPR2 deficiency [123]. A phase IIa trial studied the safety efficacy of a 16-week treatment with low-level tacrolimus in PAH patients, but the drug fell short of showing any improvement on symptom effect, exercise tolerance, RV function or serological heart failure markers [123]. However, there are mixed results in clinical trials as in some cases it has been shown to cause positive vascular remodeling properties and disease-modifying qualities in patients with severe group 1 PAH.

Chloroquine and hydroxychloroquine, which are common anti-malarial and immunomodulatory class medications, have also been tested in hopes of benefiting PAH patients due to their anti-inflammatory and anti-proliferative properties via their lysosomal activity reduction mechanisms leading to impaired autophagosome fusion with lysosomes [123]. The accumulation of impaired and ineffective autophagosome units causes the activation of apoptotic pathways and cell death [123]. Lysosomal deficiency in PAH can be advantageous by reducing BMPR2 degradation and promoting SMC apoptosis thereby effectively causing tissue remodeling [123]. The STRATOSPHERE 2 study, which is a randomized placebo-controlled phase II trial organized in the UK, evaluated chloroquine and phenylbutyrate in patients with the idiopathic or hereditary PAH variant caused by mutations in *BMPR2* in WHO functional class I-IV and on stable PAH therapy [123].

Epigenetic alterations through regulatory mechanisms in gene expression include DNA methylation, histone modification via acetylation, methylation, phosphorylation, and ubiquitination, and non-coding RNA modifications [123]. These epigenetic alterations occur without the modification and have played an important role in the development of new medications to decrease the negative impact of PAH. Olaparib, a PARP inhibitor and a chemotherapeutic agent used for ovarian, breast, and prostate cancers, was the drug centered in the OPTION trial [123]. The OPTION trial is a multicenter, open-label, phase 1B study, assessing the efficacy of Olaparib in PAH patients and its impact on hemodynamics by performing a cardiac catheterization at baseline and at 24 weeks [123]. Apabetalone, a small oral molecular inhibitor of BRD4, was studied as an open-label, single-arm, 16-week, early phase I trial with early analysis showing that it improved PVR and cardiac output in PAH patients [123].

Tyrosine kinase inhibitors such as imatinib and seralutinib are therapies that specifically target growth factors with imatinib being the first predominantly non-vasodilatory drug to be tested in PAH patients and helped improve pulmonary hemodynamics [124]. Both imatinib and seralutinib have anti-proliferative properties with seralutinib being administered in an inhaled route of administration causing it to have a more targeted approach to combating PAH [124]. Imatinib was initially used for the treatment of chronic myeloid leukemia and targets not only the BCR-ABL receptor but also targets PDGFR and c-KIT and through the IMPRES phase III clinical trial showed a reduction in pulmonary arterial pressures, PVR, and improved exercise tolerance even in patients with poor prognostic factors with severe PAH [124].

Seralutinib, as mentioned before, is administered as an inhalant and acts directly on the pulmonary vasculature with preclinical studies in animal models showing a reduction in pulmonary artery wall thickness, decreased SMC proliferation and lowered inflammation in lung tissues while other surprising findings include a reduction in PAP and right ventricle (RV) hypertrophy which alludes to its beneficial tissue remodeling in the pulmonary vasculature [124]. Two landmark studies, namely the TORREY and PROSERA clinical trials, investigated the beneficial outcomes of seralutinib in patients with PAH; however, both studies showed some negative side effects of the medication, namely temporary transaminitis, headaches, and cough, leading to some hesitancy from the experimental clinical designers of these studies [124].

Cytokines like tumor necrosis factor α (TNF-α) and interleukin 6 (IL-6) also play a significant role in chronic inflammation especially in both idiopathic and heritable PAH with studies showing that higher levels of these cytokines have negative and poor prognostic outcomes [123]. The ongoing SATISFY-JP trial is assessing the IL-6 receptor antagonist, tocilizumab, and the impact it has on group 1 PAH patients whilst measuring changes in PVR and six-minute walk distance (6MWD) from baseline to 24 weeks [123]. Rituximab, which is a target of CD20 receptors present on B-cells and is implicated in autoimmune conditions such as systemic sclerosis and SLE, was also utilized in many clinical trials to gauge the improvement of pulmonary hemodynamics in patients with connective tissue variant PAH, but results did not show a significant improvement in the primary endpoint of 6MWD or change in PVR after 24 weeks [123].

Neutrophils release elastase, degrading the extracellular matrix which leads to subsequent pulmonary vasculature tissue remodeling via the migration and proliferation of SMCs and fibroblasts [123]. Patients with PAH have elevated levels of neutrophil elastase and reduced levels of its endogenous inhibitor, elafin [123]. Elafin (Tripelestat) was studied during a small phase II trial in order to investigate suitability for patients with PAH [123]. ZMA001, which is a monoclonal antibody shows great promise in its phase I trial by preventing monocytes and macrophages from migrating and contributing pulmonary vascular remodeling [123].

The neurotransmitter serotonin plays an intricate role in proliferation and contraction of SMCs in the pulmonary vasculature and the enzyme tryptophan hydroxylase 1 (TPH1) facilitates the conversion of tryptophan to serotonin [123]. This enzyme is overexpressed in the endothelial cells lining the pulmonary vasculature in patients with PAH [123]. Lastly, a drug called rodatristat, which is a potent peripheral inhibitor of TPH1, was investigated in the ELEVATE 2 trial as a viable option to decrease SMC proliferation and contraction of the pulmonary vasculature [123]. However, this randomized, double-blind, multicenter study evaluating the changes in PVR as the primary endpoint, while secondary endpoints such as 6MWD, NT-proBNP levels, and echocardiographic indicators of disease progression over 24-week period showed disappointing results when compared to the placebo [123]. Furthermore, results from the ELEVATE 2 trial showed increases in NT-proBNP concentration, and right atrial pressures, along with a decrease in stroke volume and cardiac index, concluding that serotonin also contributes to myocardial contractility and thus a reduction in this crucial neurotransmitter can also have detrimental impact on cardiac function [123]. A comprehensive list (Table 4) of available therapeutic modalities and management strategies have been provided to summarize established treatments, and options that failed to proceed to final phases of clinical research but showed initial beneficial profiles.

### 10.3. Group II Pulmonary Hypertension Associated with Left-Sided Heart Disease

The therapeutic modalities that are effective in group 2 PH include those targeting the management and treatment of left-sided heart failure via the implementation and initiation of a specific, evidence-based treatment plan consisting of a combination of medications termed guideline-directed medical therapy (GDMT). A majority of left-sided heart failure cases can broadly be separated into HFpEF and HFrEF. The GDMTs that optimize and improve heart function in patients with HFrEF include angiotensin receptor-neprilysin inhibitors (ARNI), β-blockers, sodium-glucose cotransporter 2 inhibitors (SGLT2i), mineralocorticoid receptor antagonists (MRA), and loop diuretics as needed for patients that are fluid-overloaded making volume management important in the treatment of group 2 PH [125]. GDMTs utilized in HFpEF include SGLT2i, MRA, and loop diuretics [125]. Sodium and fluid restriction, and management of hypertension are also factors that can improve signs and symptoms in group 2 pulmonary hypertension [71].

Epoprostenol is a synthetic analogue of a naturally occurring prostacyclin (prostaglandin I_2_) which causes direct vasodilation of the pulmonary and systemic arterial vasculature and inhibition of platelet aggregation. Epoprostenol was utilized in the Flolan International Randomized Survival Trial (FIRST) study on patients with class IIIB/IV HF and decreased LVEF and demonstrated a significant increase in cardiac index (CI), decreased PAWP, and decreased systemic vascular resistance (SVR) [126]. Later during the early phases of the trial, the FIRST study had to be terminated due to increased mortality in the treatment arm [122]. Thus, the FIRST study showed increased mortality with epoprostenol despite improved hemodynamics [126]. Accordingly, PAH-specific therapies are not recommended in Group 2 PH outside of clinical trials [2].

The pathophysiological justification for the contraindication of epoprostenol in the FIRST trial when providing therapies to patients with group 2 PH-LHD was centered around the negative impact that flash pulmonary edema has [122]. The implication that epoprostenol has on this group is due in part to the excessive flid accumulation that would already exacerbate a hemodynamic constrained left heart. Thus, even though initial cardiac metrics such as an increased CI, decreased PAWP, and decreased SVR were beneficial for symptomatic relief these effects were short lived and overshadowed by the increased mortality secondary to elevated left-sided filling pressures.

Sildenafil has had some success in improving hemodynamics which led to symptom improvement in patients with group 2 PH associated with left-sided heart disease. Sildenafil helped improve patient’s cardiorespiratory exercise fitness capacity (VO_2_) in those individuals with systolic heart failure at a cost of increasing headaches in the treatment group [122]. Another study looked at patients with an EF ≤ 40% and on standard medical therapy and when combined with sildenafil those patients had significant improvement in VO_2_ and pulmonary artery systolic pressure (PASP) [127]. Guazzi et al. investigated patients with HFpEF and a systolic pulmonary artery pressure (sPAP) > 40 mm Hg and with the addition of sildenafil it provided significant improvement in mPAP, PAWP, PVR, right ventricular end diastolic pressure (RVEDP), CI, and PFT in the treatment group [128].

The surgical interventions available to patients with group 2 PH associated with left-sided heart disease include valve replacements, assisted devices, and bypass grafting [122]. Minimally invasive catheterization techniques and electrophysiology studies with subsequent arrhythmia ablations are also viable options in patients with group 2 PH with left-sided heart disease [122]. AS, which was previously mentioned as a procedure exclusive to group 1 PH patients, paradoxically worsens group 2 PH in patients with HFpEF by increasing preload to the left ventricle [122]. An interesting key prognostic metric in group 2 PH is the RV-PA coupling utilized in transcatheter valve interventions as it can predict poor outcomes in transcatheter aortic valve implantation (TAVI), transcatheter edge-to-edge repair (TEER), and tricuspid therapies [129].

Newer studies currently being investigated include the APEX study which is looking into the efficacy of a long-acting, Fc-relaxin-related fusion protein agonist for treating pulmonary hypertension secondary to heart failure with preserved ejection fraction (PH-HFpEF). Currently, the APEX study is a multi-center study design with initial promise related to the drug reportedly showing, in its earlier phases, that a single intravenous dose of TX_45_ improves both left heart function and pulmonary hemodynamics. This current APEX study is still in the beginning phases of the trial and is pending final commencement to gather data and analyze as such for any beneficial responses.

### 10.4. Group III Pulmonary Hypertension Associated with Lung Disease

The management of group 3 pulmonary hypertension is centered around the mitigation of disease progression and complications related to the underlying intrinsic lung pathologies seen in COPD, ILD, CF, and BPD [71]. It is vital that patients with hypoxemia receive long-term oxygen therapy (LTOT) as a recent prospective study investigating COPD patients on LTOT showed an improvement of mean PAP and prevented worsening of PH [114]. Supplemental oxygen or non-invasive positive pressure ventilation, and in certain circumstances when both are utilized due to worsening hypoxia and an increase in oxygen demand, can assist patients in decreasing symptom exacerbations such as shortness of breath and dyspnea upon exertion. The goal of supplemental oxygen and non-invasive positive pressure ventilation in these circumstances is to maintain an SpO_2_ ≥ 90% and PaCO_2_ ≤ 40 mm Hg [71]. 

Inhaled treprostinil, which is a prescription medication utilized in the treatment of PAH and PH associated with interstitial lung disease (PH-ILD), has also been studied to be effective in the treatment of PH-ILD [71]. Targeted pulmonary vasodilator therapy is reserved for selected patients. Inhaled treprostinil improved exercise capacity and reduced clinical worsening in patients with PH associated with interstitial lung disease in the INCREASE trial [130]. Sildenafil has shown mixed results in interstitial lung disease populations, with symptomatic improvement in selected subgroups but no consistent effect on disease progression [131,132].

The Sildenafil Trial of Exercise Performance in Idiopathic Pulmonary Fibrosis (STEP-IPF) study was a landmark clinical trial that showed sildenafil treatment in idiopathic pulmonary fibrosis (IPF) patients helped vasodilate well-ventilated areas of the lung, thereby improving gas exchange [131]. The STEP-IPF did not meet the primary objective of improving the six-minute walk distance (6MWD) test in patients with advanced IPF but helped relieve dyspnea and quality of life [114]. A second trial investigating the efficacy of sildenafil in addition to pirfenidone, an anti-fibrotic medication, on patients with advanced IPF and increased risk of PH with a mPAP ≥ 20 mm Hg, PAWP < 15 mm Hg, or evidence of intermediate or high probability of PH on echocardiogram showed no significant primary endpoint of disease progression measured by 6MWD, respiratory-associated hospitalization, or all-cause mortality [132].

The initiation of a new investigation looking at the impact of pulsed inhaled NO and its potential therapeutic benefits in patients with IPF that are at risk of PH has shown promise with early results showing improved physical activity with this intervention [133]. Another study is currently looking into the benefits of pulsed inhaled NO in PH associated with IPF patients who are already on LTOT [134]. Thus, pulsed inhaled nitric oxide remains investigational in hopes of discovering future therapeutic benefits.

### 10.5. Group IV Pulmonary Hypertension: Chronic Thromboembolism (CTEPH) and Obstruction

Chronic thromboembolic pulmonary hypertension (CTEPH) is a potentially curable form of PH. Lifelong anticoagulation is mandatory, and all patients should be evaluated at specialized centers. Medical treatment for group 4 pulmonary hypertension associated with the presence of chronic thromboembolic events includes riociguat, which is a soluble guanylate cyclase stimulator acting in similar fashion to the NO pathway thereby increasing the production of cGMP [114]. Riociguat (Adempas ™) was also investigated as a therapeutic option in patients with idiopathic interstitial pneumonia (IIP)-related PH. The Riociguat for Idiopathic Interstitial Pneumonia-associated Pulmonary Hypertension (RISE-IIP) trial evaluated riociguat as a hopeful option for management of precapillary PH but failed to show improvement in 6MWD, and was found to be detrimental in causing serious adverse events including the worsening of ILD and pneumonia, and increasing mortality, which led to the early termination of the study due to patient complications [135].

A special subset of patients that have symptomatic chronic thromboembolic pulmonary disease without PH at rest may benefit from pulmonary endarterectomy (PEA) which is a surgical procedure offered to eligible patients to help improve prognosis [72]. The European Society of Cardiology (ESC) guidelines strongly recommend PEA as a viable option and treatment choice for group 4 CTEPH. The location of thromboembolic disease in the pulmonary vasculature is also important in surgical planning as proximal lesions in the main, lobar or segmental arteries are amenable to PEA, whereas distal disease from mid-segmental and subsegmental branches is more complicated [72]. Sickle cell disease has also been known to cause group 4 PH, and lifelong anticoagulation to prevent CTEPH is practiced along with the prevention of hypoxemia, hypothermia, and acidosis [73].

In the setting of various contraindications, or if patients are not candidates for PEA, treatment with balloon pulmonary angioplasty (BPA) can be an option [73]. BPA involves a minimally invasive procedure utilizing balloons on catheters to open narrow or occluded pulmonary arteries, thereby restoring blood flow, relieving shortness of breath, improving exercise tolerance, and overall perfusion-oxygen status impacting cardio-pulmonary physiology. A study conducted to investigate the potential benefits of BPA in patients with group 4 CTEPH showed that patients undergoing BPA had significant reductions in PVR [136].

### 10.6. Group V Pulmonary Hypertension: Multifactorial Mechanisms

Group 5 PH includes heterogeneous conditions with unclear or multifactorial mechanisms. Management focuses on treatment of the underlying disorder and supportive care, with pulmonary vasodilators considered on an individualized, off-label basis in specialized settings [71,73]. The treatment of the underlying disease state remains the primary management and treatment strategy of group 5 PH associated with unclear or multifactorial mechanisms. Patients with hemolytic anemias complicated by group 5 PH can be given supplemental oxygen to reverse hypoxemia and prevent negative consequences of pulmonary vascular remodeling and stenosis [73]. Diuretics have been utilized to treat volume overload but should be given sparingly as this can cause sickling in patients with sickle cell disease (SCD) [73]. 

The usage of hydroxyurea in SCD patients has been shown to be beneficial by decreasing sickle cell hemoglobin polymerization, reducing hemolysis and frequency of acute chest syndrome, and vaso-occlusive crises while lowering rates for hospitalization and mortality [73]. Allogeneic hematopoietic stem cell transplant and JAK inhibitors to treat myeloproliferative disorders have been shown to improve PH, resulting in improvement in right ventricular function and PASP, which has been verified by echocardiogram and associated with significant reduction in NT-proBNP while increasing NO concentration [73,137,138]. For patients with extramedullary hematopoiesis hematological disorders, external beam radiotherapy focused on to the thorax has been used as a treatment modality with results showing improvement in pulmonary artery pressures [139].

### 10.7. Advanced Therapies and Transplantation

Transplantation represents the definitive therapy for patients with advanced PH who fail to stabilize despite optimized medical treatment. In PAH, bilateral lung transplantation is most commonly performed, whereas heart–lung transplantation is reserved for select patients with irreversible left-sided or biventricular failure. Early referral for transplant evaluation is emphasized in contemporary guidelines to preserve candidacy before irreversible end-organ dysfunction occurs [2].

## 11. Conclusions

Pulmonary hypertension is a debilitating disease that, without adequate and urgent medical treatment, can cause multi-organ failure and even death. It is estimated that approximately 50–70 million people worldwide, or close to 1% of the total world population, suffer from the wide spectrum of complications related to pulmonary hypertension. The five main groups of pulmonary hypertension are group I pulmonary arterial hypertension (PAH), group II pulmonary hypertension associated with left heart disease (PH-LHD), group III pulmonary hypertension associated with lung disease (PH-LD), group IV pulmonary hypertension associated with chronic thromboembolic events (CTEPH), and lastly, group V pulmonary hypertension which encompasses pulmonary hypertension that has developed from other causes and etiologies. Each group of pulmonary hypertension involves a wide variety of complex molecular mechanisms and pathophysiologic sequences in addition to the diagnostic imaging modalities utilized to better assess prognostic factors impacting both morbidity and mortality in each respective group. Since group I PAH has been the difficult variant to manage and treat and often presents with an abundance of possible causes, new and modern drug investigational trials including the ground-breaking creation of sotatercept has thrusted this disease modification to the forefront.

While other groups of pulmonary hypertension such as group II PH-LHD, group III PH-LD, and even group IV CTEPH all have treatment management strategies via controlling the underlying disease (i.e., left heart disease in group II and lung disease in group III), group I PAH treatment modalities are still being investigated and newer clinical trials and studies are being pursued. It is imperative for the demographic population stricken with pulmonary hypertension to find refuge in new scientific advancements pertaining to the discovery of new cost-effective medications as well as the development of diagnostic imaging modalities that can accurately predict future prognostic indications in order to prevent poor health outcomes. This comprehensive review establishes a profound understanding of and thorough foundation to the field of pulmonary hypertension and all its different groups and subtypes in hopes of educating the readers of the breadth of this disease process. It provides clinical analysis and updated relevant clinical trials with the goal of disease management and pertinent therapeutic modalities.

## Figures and Tables

**Table 1:** The 16 common genes associated with the genetic and molecular features of PAH.

Gene	Mode of Inheritance	Molecular Mechanism	Functional Role (s)
*BMPR2*	AD	Haploinsufficiency	Cell growth, division, & death
*EIF2AK4*	AR/AD	Loss of Function	Protein kinase sensor in detecting amino acid starvation in cells; role in ISR
*ACVRL1*	AD	Haploinsufficiency	Role in angiogenesis & vascular integrity maintenance
*TBX4*	AD	NK	Development of limbs, lungs, & umbilicus
*GDF2*	AD/AR	Haploinsufficiency	Implicated in bone development, angiogenesis, & cholinergic differentiation of neurons in the CNS
*SOX17*	AD	NK	Formation of endoderm, CV system, & other derived organs
*ENG*	AD	NK	Provides the genetic instructions for creation of endoglin protein. Important in angiogenesis. Mutation can lead to HHT
*KCNK3*	AD	Haploinsufficiency	Regulation of cellular resting membrane potential; vitally important in PASMCs
*ABCC8*	AD	Haploinsufficiency	Aids in design of SUR1 protein; component of K-ATP channel
*ATP13A3*	AD	NK	Encodes polyamine transporter protein; functions to aid in endocytosis-dependent polyamine transport
*SMAD9*	AD	Haploinsufficiency	Encodes protein involved in BMP signaling pathway; involved in developmental processes such as cell growth, differentiation, & apoptosis.
*AQP1*	AD	NK	Encodes Aquaporin 1 protein involved in abnormal PASMC proliferation & migration
*CAV1*	AD	Gain of Function; Dominant Negative	Mutations can lead to loss of Caveolin-1 protein with dysregulation of caveolae on cell membranes of pulmonary blood vessels
*BMP10*	AD	NK	Important in vascular homeostasis signaling pathway
*SMAD4*	AD	NK	Mutation can lead to abnormal proliferation of smooth muscle & endothelial cells in the pulmonary arteries
*SMAD1*	AD	NK	Facilitates the cellular signaling pathway important for BMPR

**AD** = autosomal dominant; **AR** = autosomal recessive; **NK** = not known; **ISR** = integrated stress response; **CNS** = central nervous system; **CV** = cardiovascular; **HHT** = hereditary hemorrhagic telangiectasia; **PASMCs** = pulmonary artery smooth muscle cells; **SUR1** = sulfonylurea receptor 1; **K-ATP** = potassium adenosine triphosphate; **BMP** = bone morphogenetic protein; **BMPR** = bone morphogenetic protein receptors.

**Table 2:** Categories of pulmonary arterial hypertension (PAH) ^1^.

Section 6.1.1	Idiopathic PAH (IPAH)
Section 6.1.2.	Heritable PAH (HPAH)
Section 6.1.3.	Connective Tissue Disease (CTD)-associated PAH
Section Systemic Sclerosis	Systemic Sclerosis (SSc)
Section Systemic Lupus Erythematosus	Systemic Lupus Erythematosus (SLE)
Section Mixed Connective Tissue Disease	Mixed Connective Tissue Disease (MCTD)
Section Rheumatoid Arthritis	Rheumatoid Arthritis (RA)
Section Sjögren’s Syndrome	Primary Sjögren Syndrome (pSS)
Section Idiopathic Inflammatory Myopathies	Idiopathic Inflammatory Myopathies
Section 6.1.4	Pulmonary Arterial Hypertension Associated with Congenital Heart Disease
Section 6.1.5	Drug-and-Toxin-Induced PAH
Section 6.1.6	HIV-associated PAH
Section 6.1.7	PAH associated with Portal Hypertension
Section 6.1.8	Persistent Pulmonary Hypertension of the Newborn
Section 6.1.9	Infection-Induced PAH

^1^ Ref. [41].

**Table 3:** Advantages and disadvantages of various imaging studies for pulmonary hypertension.

Imaging Study	Advantages	Disadvantages
Echocardiogram	Non-invasive Fast	Operator dependent Effect of body habitus
Right heart catheterization	Can directly measure mPAP Gold standard for diagnosis of PH	Invasive with possible complications (bleeding, perforation, arrhythmias) Exposure to ionizing radiation Contrast exposure
CT coronary angiography	Can diagnose LMCA compression, a complication of PH	Exposure to ionizing radiation Exposure to contrast dye
CT pulmonary angiography	Gold standard for diagnosis of PE, which is one cause of PH	Exposure to ionizing radiation Exposure to contrast dye
Cardiac MRI	Prognostic for PH	Expensive & financially prohibitive
V/Q scan	Low risk & non-invasive Negative predictive value of 98% for CTEPH	Exposure to ionizing radiation Low specificity Difficulty with precise location of defect
PET scan	Assess RV function & predict prognosis using molecular tracer FDG Gauge metabolic alterations & myocardium tissue remodeling	Ionizing radiation exposure Expensive & financially prohibitive

**mPAP** = mean pulmonary arterial pressure; **PH** = pulmonary hypertension; **LMCA** = left main coronary artery; **PE** = pulmonary embolism; **CTEPH** = chronic thromboembolic pulmonary hypertension; **RV** = right ventricle; **FDG** = ^18^F-fluorodeoxyglucose.

**Table 4:** Therapeutic modalities and available treatment options for group 1 PAH including medications that initially showed positive outcomes but later failed final verification due to side effects profiles, amongst other negative findings.

Drug Class	Physiologic Effect	Examples of Medications
Endothelin Receptor Antagonist (ERA)	Prevents pulmonary vasculature tissue remodeling and narrowing through the inhibition of vasoconstriction, cell proliferation, and fibrosis. Reduces PVR, and improving exercise capacity.	Bosentan, Ambrisentan, Macitentan
Phosphodiesterase-5 inhibitors (PDE-5i)	Increased blood flow via vasodilation of pulmonary vessels, improved cardiac function, and reduced vascular remodeling. Blocks the degradation of cyclic guanosine monophosphate (cGMP) in pulmonary vascular smooth muscle cells.	Sildenafil, Tadalafil
Prostanoids (prostaglandins & prostacyclin analogs)	Keeps pulmonary vasculature open via vasodilation, inhibits platelet aggregation, and provides antiproliferative effects. Increases intracellular cyclic adenosine monophosphate (cAMP).	Epoprostenol, Iloprost, Treprostinil
Selective, non-prostanoid receptor (IP receptor) agonist	Vasodilation and decreasing PVR, anti-proliferation, and anti-platelet effects.	Selexipag
Soluble guanylate cyclase (sGC)	Inhibits vascular remodeling by stabilizing nitric oxide (NO)-sGC binding and directly activating sGC independent of NO. Inducing vasodilation, reducing inflammation, and inhibiting vascular remodeling.	Riociguat
Calcium channel Blockers (CCB)	Reduction in pulmonary artery pressure. Inhibit calcium influx through voltage-gated channels in vascular smooth muscle cells, causing pulmonary artery relaxation & dilation.	Nifedipine, Diltiazem, Nicardipine, & Amlodipine
Activin-signaling inhibitor	Reverses pulmonary vascular remodeling, reduces pulmonary vascular resistance, lowers pressures, and targets underlying cause of disease progression. Activin-signaling inhibitor that acts as fusion protein “ligan trap” to rebalance pro-proliferative (activin/SMAD2/3) and anti-proliferative (BMPR2/SMAD1/5/8) signaling.	Sotatercept
Calcineurin inhibitor	Reduction in autoimmune-mediated vascular remodeling. Binds to repressor, FKBP12, and prevents its interaction with the TGF-β pathway thus increasing BMPR2 signaling to restore BMPR2 deficiency.	Tacrolimus
Lysosomal inhibitors	Disrupt lysosomal degradation by inhibiting excessive cellular autophagy and restoring BMPR-2 receptor signaling. Prevent vascular remodeling associated with PAH by inducing apoptosis of pulmonary artery smooth muscle cells.	Chloroquine & Hydroxychloroquine
PARP inhibitor	Reduction in pathological remodeling of pulmonary vessels, reverse RV dysfunction, decrease inflammation, offering a potential disease-modifying therapeutic modality. Inhibits enzyme PARP1, which is overactivated by DNA damage in the damaged endothelial cells of the pulmonary vasculature and the heart.	Olaparib
Selective bromodomain & extraterminal (BET) inhibitor	Regulation of gene expression via epigenetic therapy by decreasing pulmonary vascular remodeling, and reduced inflammation leading to improved cardiac output and reduced vascular resistance. Inhibition of BRD4-dependent transcription by binding to BD2 domain.	Apabetalone
Tyrosine kinase inhibitors (TKI)	Promotes apoptosis of pulmonary artery smooth muscle cells and reverses pathologic pulmonary vascular remodeling. Primarily inhibits platelet-derived growth factor receptors (PDGFR-α, PDGFR-β) and c-KIT.	Imatinib & Seralutinib
Interleukin-6 (IL-6) receptor antagonist	Reduction in inflammation that can contribute to vascular remodeling. Humanized monoclonal antibody that binds to both soluble and membrane-bound IL-6 receptors.	Tocilizumab
Anti-CD20 Antibodies	Decrease pulmonary vascular remodeling and improve symptoms. Target & deplete CD20+ B-cells and thereby inhibit their roles in autoantibody production and autoimmune-mediated vascular damage.	Rituximab
Serine Protease Inhibitor (specifically a leukocyte elastase inhibitor)	Promotes apoptosis of overgrown smooth muscle cells, and enhances BMPR2. Anti-inflammatory and tissue-protective protein that reverses vascular remodeling by inhibiting neutrophil elastase (NE), and proteinase 3, reducing extracellular matrix degradation.	Elafin (Tripelestat)
Anti-membrane-exposed lysyl-tRNA synthetase (KARS1)	Reduction in pulmonary vascular remodeling and fibrosis. Inhibits KARS1 dependent infiltration of inflammatory monocytes and macrophages into the pulmonary vessels.	ZMA001 (Rapaprutug)
Tryptophan hydroxylase 1 (TPH1) inhibitor	Aimed at halting and reversing the pathologic remodeling of pulmonary vasculature, excessive proliferation of smooth muscle cells of PAH. Potent inhibitor of tryptophan hydroxylase (TPH) and causing reduction in peripheral serotonin (5-HT) synthesis.	Rodatristat

## Data Availability

No new data were created or analyzed in this study. Data sharing is not applicable to this article.
